# Selective Targeting of Tip Endothelial Cells as a Therapeutic Strategy for Tumor Angiogenesis

**DOI:** 10.1002/advs.202512975

**Published:** 2026-02-15

**Authors:** Byoungmo Kim, Ha Kyeong Lee, Zulfikar Azam, Jeong Uk Choi, Riajul Wahab, Na Kyeong Lee, Yoon Gun Ko, So‐Young Choi, Se‐Ra Lee, Wan Seob Shim, Taeeung Kim, In‐San Kim, Farzana Alam, Sang Yoon Kim, Seong Who Kim, Youngro Byun, Taslim A Al‐Hilal

**Affiliations:** ^1^ College of Pharmacy and Research Institute of Pharmaceutical Sciences Seoul National University Seoul Republic of Korea; ^2^ Department of Molecular Pharmaceutics and Biomedical Engineering University of Utah Salt Lake City Utah USA; ^3^ College of Pharmacy Kyung Hee University Seoul Republic of Korea; ^4^ College of Pharmacy and Research Institute for Drug Development Pusan National University Busan Republic of Korea; ^5^ Pharosgen Co. Ltd Seoul Republic of Korea; ^6^ New Drug Development Center Osong Medical Innovation Foundation 123 Osongsaengmyeong‐ro Heungdeok‐gu Cheongju Chungbuk Republic of Korea; ^7^ KU‐KIST Graduate School of Converging Science and Technology Korea University Seoul Republic of Korea; ^8^ Department of Pharmacology and Toxicology University of Utah Salt Lake City Utah USA; ^9^ Asan Medical Center University of Ulsan College of Medicine Seoul South Korea

**Keywords:** angiogenesis, monoclonal antibodies, tip cells, tumor therapy

## Abstract

Tip endothelial cells (^Tip^EC), the leading edge of angiogenic sprouts, are essential for pathological neo‐vascularization but remain difficult to target due to the lack of specific druggable markers. Here, we identify Doppel as a selective and druggable regulator of endothelial tip cell function. Doppel expression enhances ^Tip^EC selection, directional migration, and regulates tip‐stalk cell dynamics by spatially controlling VEGFR2/Dll4/Src pathway. Genetic ablation of *PRND* (Doppel) reduces tip cell formation without affecting the stalk cells (^Stalk^ECs) number in tumors, indicating its selective role in ^Tip^ECs. Importantly, depletion of ^Tip^ECs using the first‐in‐class monoclonal antibodies against a highly conserved WQF‐motif of Doppel robustly decreased the growth of tumors by selectively downregulating VEGFR2+ ^Tip^ECs but not ^Stalk^ECs. These findings position Doppel as a tumor ^Tip^EC‐specific, druggable target that may offer a new avenue to enhance and refine anti‐angiogenic therapies in cancer treatment.

## Graphical abstract

Doppel protein is selectively expressed in tip endothelial cells within the tumor vasculature, where it promotes tip cell motility and stabilizes the tip cell phenotype. Targeting Doppel with monoclonal antibodies disrupts this stabilization, impairs angiogenic sprouting, and reduces tumor angiogenesis, offering a selective and druggable switch for therapeutic vascular targeting.

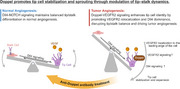

## 1 Introduction

Sprouting angiogenesis is tightly regulated by a balance of tip and stalk endothelial cells (ECs), i.e., tip‐stalk specification in response to pro‐angiogenic factors [1]. Tip endothelial cells (^Tip^ECs) extend dynamic filopodia to navigate toward angiogenic gradients and direct vascular sprouting, while stalk cells (^Stalk^EC) proliferate to form the vascular structure. Tip‐stalk cell selection is a highly dynamic process, with endothelial cells transitioning between these states to reflect minute changes in the microenvironment [1, 2]. The increased presence of ^Tip^ECs in tumor‐associated vasculature suggests an active yet dysregulated angiogenic process that supports tumor growth and progression [3, 4]. However, due to the plasticity of tip‐stalk identity, identifying a definitive molecular marker for ^Tip^ECs remains challenging [5]. While several molecular regulator of ^Tip^ECs function have been identified, no molecular regulators has emerged as a druggable target due to their widespread role in physiological and developmental angiogenesis.

Doppel (*PRND)*, a paralog of the prion protein (*PrP*), has recently emerged as a candidate molecule associated with pathological angiogenesis. Originally studied in the context of testicular function and neurodegenerative disorders [6, 7, 8, 9], Doppel has since been implicated in vascular malformations in hereditary hemorrhagic telangiectasia [10] and the aberrant vasculature of multiple tumor types [11, 12, 13, 14, 15, 16]. Our previous work identified Doppel as a coreceptor for multiple surface receptor kinases, particularly VEGFR2, driving aggressive angiogenesis in tumor ECs [11]. Interestingly, Doppel expression appears to be limited to pathological angiogenesis, except for neonatal brain development [17, 18]. This suggests that Doppel could act as a “gateway switch,” tipping the balance from regulated to unregulated angiogenesis.

In this study we redefine the role Doppel has in angiogenesis in the context of ^Tip^EC dynamics. We demonstrate that Doppel expression promotes ^Tip^EC selection, enhances directional migration along VEGF gradients, and stabilizes the ^Tip^EC phenotype. Finally, we establish that Doppel serves as a potential ^Tip^EC‐specific, druggable target using anti‐Doppel monoclonal antibodies in angiogenic tumors.

## 2 Results

### 2.1 Doppel Marks a Distinct Subpopulation of Tip Endothelial Cells in Tumors

**FIGURE 1:**
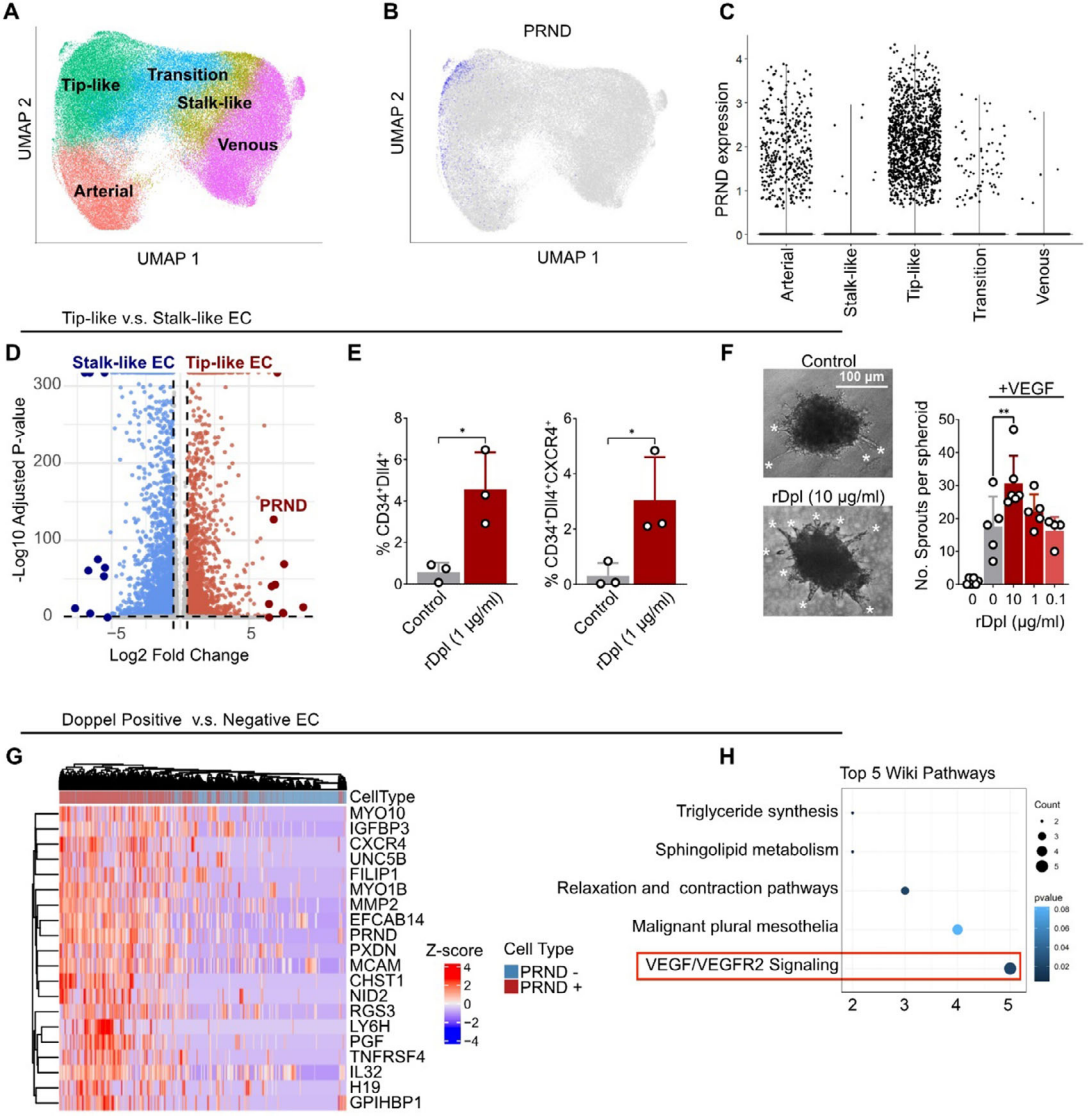
PRND is selectively expressed in tip endothelial cells (^Tip^EC) and promotes tip cell–like behavior. (A) UMAP projection of tumor‐associated endothelial cells (ECs), colored by inferred EC subtypes: arterial, venous, stalk‐like, tip‐like, and transition. (B) Overlay of *PRND* expression on the UMAP shows selective enrichment in ^Tip^EC. (C) Violin plot showing *PRND* expression across EC subtypes, with highest level detected in ^Tip^EC. (D) Volcano plot comparing gene expression between tip‐like and stalk‐like ECs. *PRND* is among the most significantly upregulated genes in tip‐like ECs. Genes with adjusted *p* < 0.05 and Log2 fold change > 0.25 were considered differenially expressed. (E) Quantification of double (CD34^+^Dll4^+^) and triple (CD34^+^Dll4^+^CXCR4^+^) gated ^Tip^EC after 24 h treatment with recombinant Doppel (rDpl, 1 µg mL^−1^) in HDMECs. Dots represent independent experiments, *n* = 3 per condition. Two‐tailed Student's *t*‐test; *p* ^*^< 0.05. (F) Representative images of VEGF‐induced endothelial sprouting from HUVEC spheroids treated with increasing concentrations of rDpl. White asterisks indicate HUVEC sprouts. Scale bar, 100 µm. Right panel: Quantification of sprouts per spheroid. *n* = 5 experimental replicates; 40–50 spheroids were counted per experiment. See Also Figure S2C. Statistical analysis was performed using one‐way ANOVA followed by Tukey's multiple‐comparison test; *p* ^**^< 0.01, nonsignificant comparisions not shown. (G) Heatmap of the top 20 differentially expressed genes (DEGs) between *PRND* ^+^ and *PRND* ^−^ ECs. A total of 1500 randomly selected cells per group were analyzed. Rows represent genes; columns represent individual cells. Expression values were log‐normalized and Z‐scored. Color scale: red = high expression; blue = low expression. (H) WikiPathways enrichment analysis of DEGs in *PRND* ^+^ versus *PRND* ^−^ ECs, highlighting VEGF/VEGFR2 signaling as the top enriched pathway. Dot size indicates gene count; color represents adjusted *p*‐value. All data are presented as mean ± standard deviation (SD).

To investigate the ^Tip^EC‐specific role of Doppel in tumors, we analyzed publicly available single‐cell RNA sequencing (scRNA‐seq) datasets of tumor endothelial cells [4]. ECs were clustered and annotated using the original metadata (Figure 1A) and categorized into five endothelial subtypes: arterial endothelial cells, tip‐like cells, transitional cells, stalk‐like cells, and venous endothelial cells. Density mapping of canonical tip cell markers KDR, Dll4, ESM1, ANGPT, APLN, and CXCR4 confirmed accurate annotation of endothelial subtypes (Figure S1A).

Density mapping of *PRND* (the gene encoding Doppel) on the UMAP projection revealed specific enrichment of Doppel expression within the tip‐like cell compartment (Figure 1B). Notably, *PRND*‐expressing cells were concentrated in the upper‐left region of the density plot, suggesting that Doppel marks a highly distinct endothelial subpopulation even within the ^Tip^EC population. Violin plot analysis further confirmed that *PRND* ^+^ cells were significantly enriched within the tip‐like endothelial population compared to other endothelial subtypes (Figure 1C), reinforcing the hypothesis that Doppel functions as a key regulator of ^Tip^EC behavior.

### 2.2 Doppel Induces a Tip Cell Phenotype in Endothelial Cell Cultures and Activates VEGFR2 Signaling

Building on these findings, we examined differential gene expression between ^Tip^ECs and ^Stalk^ECs to assess Doppel's involvement in tip‐stalk dynamics. Volcano plot analysis revealed that *PRND* ranked as the seventh most significantly upregulated gene in ^Tip^ECs relative to ^Stalk^ECs (Figure 1D). We next sought to functionally validate the role of Doppel in driving ^Tip^EC identity by treating normal endothelial cell lines with recombinant Doppel (rDpl) protein. Previous studies have demonstrated that Doppel colocalizes with VEGFR2 and enhances VEGFR2 signaling in endothelial cells [11, 18]. To confirm that rDpl treatment mimics the activity of membrane‐expressed Doppel, we labeled rDpl with Cy5.5 and confirmed its binding to the endothelial cell surface via flow cytometry (Figure S2A). Immunoprecipitation assays further validated that rDpl interacts directly with VEGFR2 (Figure S2B).

Using this system, we then assessed whether surface‐expressed Doppel could promote a tip‐like phenotype in endothelial cultures. CD34 expression, a recognized marker of ^Tip^ECs in 2D cultures, was analyzed along with additional ^Tip^EC markers Dll4 and CXCR4 in endothelial cells to assess tip‐like identity. Flow cytometry analysis revealed that treatment of rDpl led to a significant expansion of CD34^+^ endothelial subpopulation in both human umbilical vein endothelial cells (HUVECs) and human dermal microvascular endothelial cells (HDMECs) (Figure S1B) as well increased proportions of the tip cell marker gated subpopulations Dll4^+^, CD34^+^Dll4^+^, and CD34^+^Dll4^+^CXCR4^+^ in HDMECs (Figure 1E). Similarly, rDpl treatment also enhanced VEGF‐induced sprouting in HUVEC spheroids (Figure 1F; Figure S2C). Collectively, these results suggest that surface‐expressed Doppel induces a ^Tip^EC‐like phenotype in vitro.

To gain mechanistic insight of Doppel function in ^Tip^ECs, we stratified the endothelial cells from the single cell RNA sequencing dataset into *PRND^+^* and *PRND^−^* groups. Differential gene expression (DEG) and WikiPathways enrichment analysis comparing *PRND* positive to *PRND* negative endothelial cells revealed that Doppel expression was strongly associated with VEGF/VEGFR2 signaling pathways (Figure 1G,H; Figure S3A), consistent with our previous findings [11, 18]. To experimentally confirm this association, we assessed VEGFR2 phosphorylation in HUVECs following Doppel expression. Both transient transfection with Doppel‐cDNA (Figure S3B) and treatment of serial concentrations of rDpl (Figure S3C) resulted in an enhanced VEGFR2 response to VEGF gradients. Furthermore, HUVEC cells stably transfected with Doppel (Hu^Dpl^), described previously [11], maintained elevated VEGFR2 phosphorylation levels even at low VEGF concentrations (Figure S3D). Together, these results suggest that Doppel potentiates VEGFR2 activation and lowers the threshold for VEGF‐mediated signaling in ^Tip^ECs.

### 2.3 Doppel Increases Filopodia Extension and Motility in ^Tip^ECs

**FIGURE 2:**
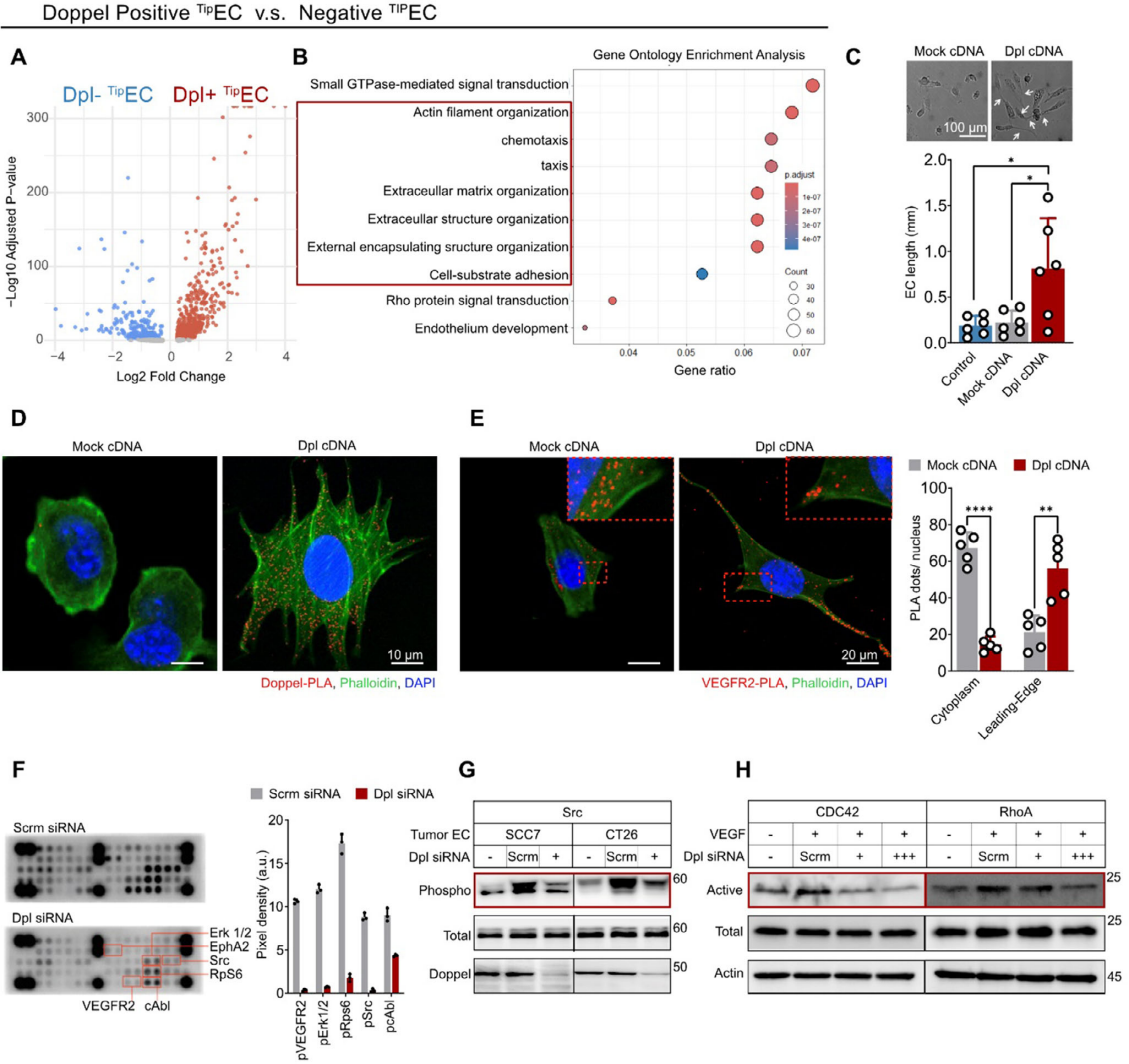
Doppel promotes tip cell morphology and cytoskeletal remodeling. (A) Volcano plot showing differentially expressed genes (DEGs) between *PRND* ^+^ versus *PRND* ^−^ within the ^Tip^EC population. Genes with adjusted *p* < 0.05 and Log2 fold change > 0.25 were considered significant. (B) Gene Ontology enrichment analysis of DEGs in *PRND* ^+^ versus *PRND* ^− Tip^ECs. Dot size indicates gene count; color represents adjusted *p‐*value. Pathways related to actin cytoskeleton and cell motility are highlighted. (C) Quantification of EC length in HDMECs transfected with either mock‐ or Doppel‐constructs. Doppel‐transfected cells exhibit elongated morphology and filopodia‐like protrusions., *n* = 6 experimental replicates. Statistical analysis was performed using one‐way ANOVA followed by Tukey's multiple‐comparison test; *p* ^*^< 0.05, nonsignificant comparisions not shown. Corresponding images are shown in Figure S3C. White arrows indicate filopodia. Scale bar, 100 µm. (D) Representative confocal images of HDMECs transfected with Doppel cDNA or mock vector. Doppel‐PLA, red dots; phalloidin, green; DAPI, blue. Scale bar, 10 µm. (E) Representative confocal image showing subcellular distribution of VEGFR2 in Doppel‐ and mock‐transfected HDMECs. In Doppel‐expressing cells, VEGFR2 localizes toward the leading edge. VEGFR2‐PLA, red dots; Phalloidin, green; DAPI, blue. Scale bar, 10 µm. Right panel: Number of VEGFR2‐PLA dots either in cytoplasm or leading‐edge. *n* = 5 experimental replicates; 50–100 cells were counted per experiment. Statistical analysis was performed using two‐tailed Student's *t*‐test; *p* ^****^< 0.0001, *p* ^**^< 0.01. (F) RTK phospho‐array analysis of Hu^Dpl^ cells transfected with scramble or Doppel siRNA. Right panel: Quantification by pixel density. *n* = 3 experimental replicates. (G) Western blot analysis for phosphorylated and total Src in ECs isolated from SCC7 and CT26 tumors following transfection with either Doppel or scramble siRNA. Doppel protein levels confirm knockdown efficiency. (H) Western blot analysis for active and total CDC42 and RhoA in Hu^Dpl^ cells following transfection with either Doppel or scramble siRNA under VEGF stimulation. All data are presented as mean ± standard deviation (SD).

To identify the functional role of Doppel in ^Tip^ECs, we sorted only the ^Tip^ECs from the single‐cell dataset into *PRND*‐positive and *PRND*‐negative groups and performed differential gene expression analysis (Figure S4A). Volcano plot visualization of the resulting DEGs revealed that Doppel expression clearly separated the ^Tip^EC population into two transcriptionally distinct subsets (Figure 2A, Supporting information). Both Gene Ontology (GO) and KEGG pathway enrichment analysis of these DEGs showed that *PRND*‐positive cells were significantly enriched for pathways regulating actin filament organization, chemotaxis, and extracellular matrix remodeling. This suggests Doppel may play a role in promoting filopodia extension and directional migration along angiogenic gradients (Figure 2B; Figure S4B). Consistent with the transcriptomic findings, compared to vector control transfected cells, Doppel transfected ECs exhibited an elongated phenotype with prominent filopodia‐like protrusions, characteristic of activated ^Tip^ECs (Figure 2C; Figure S4C). In 3D sprouting assays, Doppel‐expressing EC spheroids also showed enhanced VEGF‐induced sprouting, further supporting its role in promoting ^Tip^EC behavior (Figure S4D). These morphological and functional changes suggest Doppel promotes ^Tip^EC behavior by enhancing cytoskeletal remodeling and migratory capacity. Notably, newly expressed Doppel localized along these protrusions, further supporting its role in guiding tip‐like extensions (Figure 2D). Given that Doppel directly interacts with VEGFR2 (Figure S1B) and increases its phosphorylation (Figure S2B), thereby acting as a key regulator of ^Tip^EC sensing of angiogenic gradients, we hypothesized that Doppel may influence the subcellular localization of VEGFR2, particularly to the leading edge of ECs in the filopodia. This was tested by visualizing VEGFR2 distribution in transfected and non‐transfected ECs. In mock‐transfected ECs, VEGFR2 broadly distributed throughout the cytoplasm, whereas in Doppel transfected ECs, VEGFR2 predominantly concentrated at the leading edge of the cells (Figure 2E). This shift in VEGFR2 localization suggests that Doppel plays a role in facilitating VEGFR2 signaling at the filopodia, thereby enhancing ^Tip^EC responsiveness to the angiogenic cues.

We then investigated kinases involved in Doppel‐mediated cytoskeletal remodeling and motility by performing a receptor tyrosine kinase (RTK) signaling antibody array (PathScan) using Hu^Dpl^ cells transfected with either scrambled or Doppel‐specific siRNA. Doppel knockdown decreased the phosphorylation of VEGFR2, EphA2, Src, ERK1/2, c‐Abl, and RpS6 – all critical regulators of angiogenesis and/or cytoskeletal dynamics (Figure 2F) [19, 20, 21]. Notably, VEGFR2 and EphA2 are well‐established upstream activators of Src kinases, which play a pivotal role in cytoskeletal reorganization and endothelial migration [19, 21, 22, 23, 24]. Consistent with these findings, Src phosphorylation was significantly reduced in tumor endothelial cells (TECs) isolated from SCC7 and CT26 tumors following Doppel depletion, reinforcing Doppel's role in Src activation within ^Tip^ECs and the tumor vasculature (Figure 2G). Additionally, Doppel silencing impaired signaling through Rho family small GTPases, CDC42 and RhoA, which are known to control actin organization, cell contractility, and adhesion dynamics (Figure 2H) [22, 25, 26, 27]. These findings are consistent with Gene Ontology enrichment analyses implicating Doppel in Rho protein signal transduction and small GTPase–mediated pathways.

### 2.4 Doppel Controls Tip‐Stalk Cell Dynamics in Sprouting Vessels via VEGFR2‐Dll4 Signaling

**FIGURE 3:**
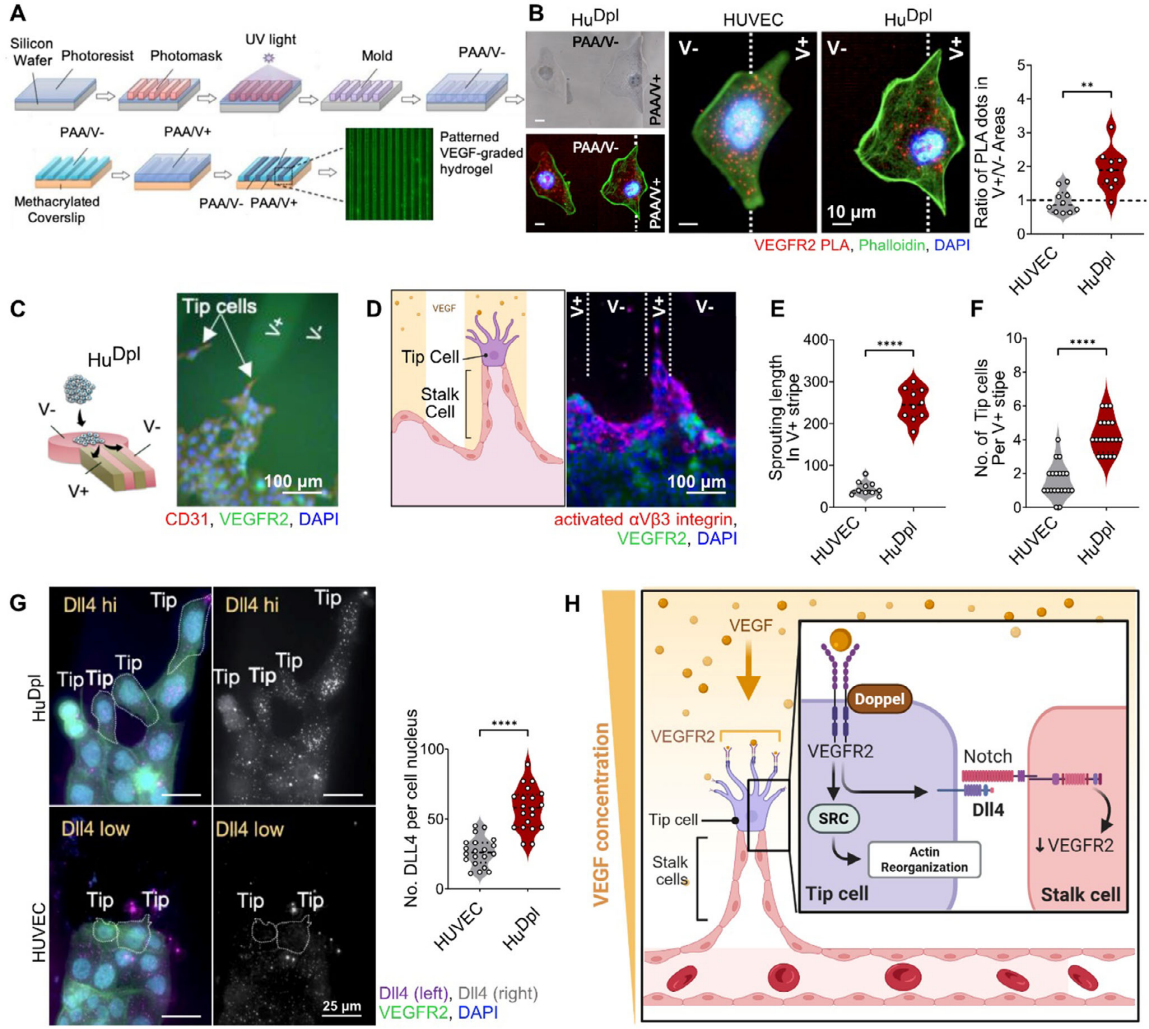
Doppel facilitates ^Tip^EC mobility and stability through VEGFR2‐mediated signaling. (A) Schematic of a bioengineering platform with alternating VEGF‐rich (V^+^) and VEGF‐depleted (V^−^) stripes for directional endothelial cell migration. (B) Brightfield and confocal microscopy images showing VEGFR2 localization relative to VEGF‐A gradients in HUVEC and Hu^Dpl^ cells. *n* = 9∼10 experimental replicates. VEGFR2, red; Phalloidin, green; DAPI, blue. Scale bar, 10 µm. Right panel: Ratio of VEGFR2‐proximal PLA dots in V^+^ and V^−^ areas for both HUVEC and Hu^Dpl^ cells. (C) Schematic of a bioengineered angiogenesis platform incorporating ECs to visualize ^Tip^EC migration and representative fluorescent image showing the sprouting of Hu^Dpl^ cells along a V^+^ stripe. CD31, red; VEGFR2, green; DAPI, blue. Scale bar, 100 µm (D) Confocal image of ^Tip^EC cells expressing activated integrin in Hu^Dpl^ sprouts. Activated αVβ3 integrein, red; VEGFR2, green; DAPI, blue. Scale bar, 100 µm (E) Quantification of sprouting length in Hu^Dpl^ versus HUVEC spheroids per V^+^ stripe. *n* = 10 sprouts. (F) Quantification of ^Tip^EC number in Hu^Dpl^ versus HUVEC spheroids per V^+^ stripe. *n* = 20 individual fields. (G) Representative confocal image of Dll4 expression in ^Tip^EC from Hu^Dpl^ versus HUVEC. Dll4, purple (left)/ grayscale (right); VEGFR2, green; DAPI, blue. Scale bar, 25 µm. Right panel: Quantification of Dll4‐positive signals per nucleus in Hu^Dpl^ versus HUVEC sprouts in the V^+^ stripes. *n* = 20 individual fields. (H) Schematic model illustrating Doppel's role in ^Tip^EC differentiation, filopodia extension and directed migration through VEGFR2 signaling. Doppel enhances VEGFR2 localization to the leading edge and activates Dll4 and downstream Src signaling pathways that stabilize tip cell identity and motility. All data are presented as mean ± standard deviation (SD). Statistical analysis was performed using two‐tailed Student's *t*‐test unless otherwise stated; *p* ^****^< 0.0001, *p* ^***^< 0.001, *p* ^**^< 0.01.

To investigate how Doppel facilitates ^Tip^EC migration toward angiogenic cues, we developed a bioengineered cell culture platform featuring alternating VEGF‐rich (V+) and VEGF‐negative stripes (V‐) (Figure 3A). When ECs were cultured on this platform, Hu^Dpl^ cells exhibited directed migration toward V^+^ regions, whereas HUVECs displayed no clear directional bias, suggesting that Doppel enhances endothelial cell responsiveness to VEGF gradients. To quantify VEGFR2 localization in relation to the VEGF gradient, we performed a proximity ligation assay (PLA). In HUVECs, VEGFR2‐PLA signals were evenly distributed across V^+^ and V^−^ interfaces (V^+^/V^−^ ratio = 0.96), despite the cells being in contact with VEGF‐rich regions, indicating a lack of polarization. In contrast, Hu^Dpl^ cells showed a marked enrichment of VEGFR2 in V^+^ regions (V^+^/V^−^ ratio = 1.91), indicating VEGFR2 redistribution at the leading edge in response to VEGF cues (Figure 3B).

We further characterized Doppel's role in ^Tip^EC sprouting by adapting the VEGF‐patterned system to model 3D angiogenic sprouting using EC spheroids (Figure 3C). Hu^Dpl^ exhibited robust sprouting in the V+ stripes (Figure S5A). These migrating Hu^Dpl^ cells displayed robust activation of αvβ3 integrin—a marker of dynamic adhesion and cellular motility [22, 28, 29] (Figure 3D) and generated substantially longer sprouts in comparison to HUVECs (Figure 3E), further highlighting Doppel's role in promoting ^Tip^EC extension. The number of ^Tip^ECs within the V+ stripes also increased in Hu^Dpl^ spheroids, indicating enhanced ^Tip^EC selection and migration (Figure 3F). Interestingly, sprouts of Hu^Dpl^ exhibited higher Dll4 expression in ^Tip^ECs compared to HUVEC sprouts (Figure 3G). This suggests that Doppel may stabilize ^Tip^EC identity through VEGFR2–Dll4–Notch signaling [30, 31].

Together, these findings suggest Doppel expression polarizes VEGFR2 signaling toward the leading edge of endothelial cells, thereby enhancing Src‐mediated actin remodeling and directional migration. Additionally, Doppel‐ driven VEGFR2 activation in ^Tip^ECs upregulates Dll4‐function, which reinforces subsequent signaling in the adjacent ^Stalk^ECs. This may disrupt physiological tip‐stalk cells dynamics and promote excessive ^Tip^EC formation in the tumor microenvironment (Figure 3H).

### 2.5 Doppel Promotes Tip Cell Sprouting in Vivo

We investigated whether Doppel expression enhances ^Tip^EC motility and number in vivo by implanting Matrigel–fibrin gels impregnated with HUVEC or Hu^Dpl^ spheroids into the flanks of SCID mice in the presence of VEGF. HUVEC spheroids implanted without VEGF were used to assess baseline vessel formation (Figure S5B). One week after implantation, HUVEC spheroids without VEGF failed to form any vascular structures. Strikingly, Hu^Dpl^ spheroids in the presence of VEGF produced a markedly different vascular bed compared to HUVEC spheroids in the same conditions. Immunostaining of the Hu^Dpl^ impregnated Matrigel revealed highly disorganized, tip cell‐rich neo‐vasculatures in comparison to the more structured vasculature observed in HUVEC spheroids (Figure S5B,C), further supporting the notion that Doppel expression enhances ^Tip^EC formation and stability in vivo. Analysis of hemoglobin content in Matrigel plugs revealed increased blood accumulation in Hu^Dpl^ plugs, suggesting that the neo‐vasculature was, to some extent, perfused and functional. (Figure S5D)

### 2.6 Doppel Knockout Reduces ^Tip^ECs but not ^Stalk^ECs in Tumors

**FIGURE 4:**
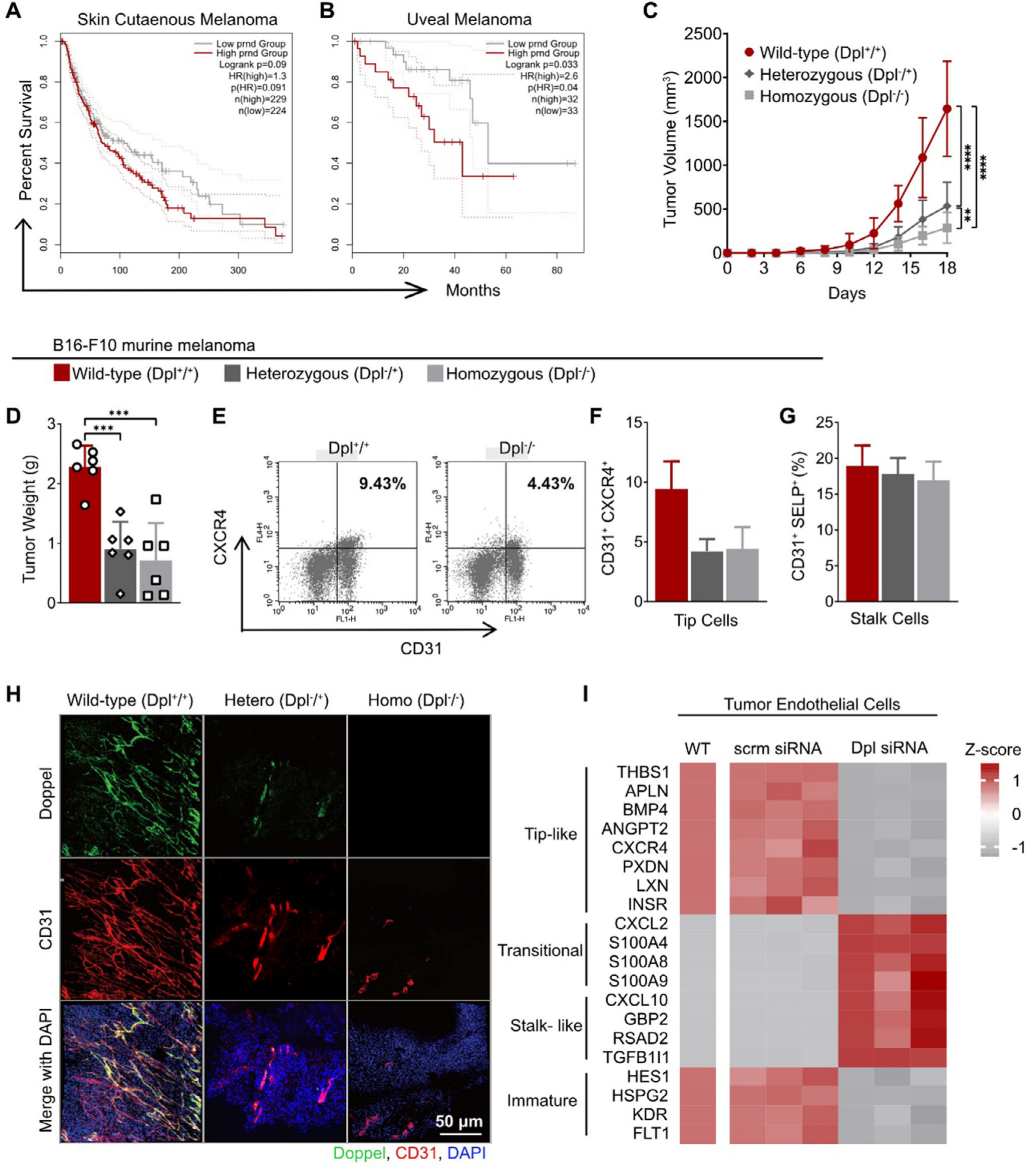
Tumor growth is dependent on Doppel expression. (A,B) Kaplan–Meier survival curves for skin cutaneous melanoma (A) and uveal melanoma (B) patients stratified by *PRND* expression levels. Analysis was performed using GEPIA2 based on TCGA datasets. (C) Tumor growth curves of B16F10 melanoma tumors in wild‐type (Dpl^+^/^+^, red), heterozygous (Dpl^+^/^−^, dark gray), and homozygous Doppel knockout (Dpl^−^/^−^, gray) C57BL/6 mice over 18 days post‐inoculation. *n* = 9 mice per group, pooled from two independent experiments. Statistical analysis was performed using two‐way ANOVA followed by Tukey's multiple‐comparison test on day 18; *p* ^**^< 0.01, *p* ^****^< 0.0001, nonsignificant comparisions not shown. (D) Tumor weights at endpoint. Dots represent indivisual tumors, *n* = 6. Statistical analysis was performed using one‐way ANOVA followed by Tukey's multiple‐comparison test; *p* ^***^< 0.001, nonsignificant comparisions not shown. (E) Representative flow cytometry plots illustrating the gating strategy used to identify CD31^+^CXCR4^+^^Tip^ECs in tumor tissues from wild‐type (Dpl^+^/^+^) and homozygous Doppel knockout (Dpl^−^/^−^) mice. (F,G) Quantification of CD31^+^CXCR4^+^^Tip^EC (F), and CD31^+^SELP^+^^Stalk^ ECs (G) from tumors wild‐type (Dpl^+^/^+^), heterozygous (Dpl^+^/^−^), and homozygous Doppel knockout (Dpl^−^/^−^) mice. (H) Representative immunofluorescent images of tumor tissues from each genotype showing vessel density and Doppel expression. Doppel, green; CD31, red; DAPI, blue. Scale bar, 50 µm. (I) Heatmap depicting transcriptomic changes in wildtype tumor endothelial cells (WT) following transfection with either Doppel or scramble siRNA. Rows represent genes; columns represent individual samples. Genes are grouped by relevant endothelial subtypes: tip‐like, transitional, and stalk‐like, and immature. Expression values were Z‐scored and color coded. Color scale: red = high expression; gray = low expression. All data are presented as mean ± standard deviation (SD).

We investigated the clinical relevance of Doppel expression in cancer by analyzing TCGA (The Cancer Genome Atlas) patient survival data. Kaplan–Meier analysis revealed that high Doppel (*PRND*) expression was associated with reduced overall survival in patients with skin cutaneous melanoma and uveal melanoma (Figure 4A, B), implicating Doppel in aggressive tumor behavior likely driven by enhanced angiogenesis. To validate this hypothesis, we generated and breeded Doppel knockout (KO) mice on an immunogenic C57BL/6 background. Consistent with previous reports, wild‐type (WT), heterozygous (Dpl^+/−^), and homozygous (Dpl^−/−^) Doppel‐KO mice developed without any overt congenital defects aside from male infertility [32, 33]. In contrast, B16‐F10 melanoma tumors implated in both Dpl^+/−^ and Dpl^−/−^ mice were significantly smaller than in WT controls (Figure 4C, D), indicating Doppel expression within the stromal compartment contributes substantially to tumor growth.

We assessed the impact of Doppel loss on the tumor endothelium by performing flow cytometry on tumor‐derived endothelial cells. Using established surface markers, we identified ^Tip^ECs (CD31^+^CXCR4^+^) and ^Stalk^ECs (CD31^+^SELP^+^CXCR4^−^) [34] (Figure 4E). Flow cytometry revealed no significant changes in ^Stalk^EC populations, whereas ^Tip^EC percentages were markedly reduced in Doppel‐KO mice (Figure 4F, G). Furthermore, CD31 immunostaining of tumor sections revealed a significant reduction in vessel density in tumors of Dpl^−/−^ mice, indicating impaired vessel sprouting in the absence of Doppel (Figure 4H). Finally, we defined the transcriptional consequences of Doppel depletion in tumor endothelial cells. RNA sequencing revealed that loss of Doppel selectively downregulated tip and immature endothelial cell–associated genes, while expression of transitional and stalk markers increased (Figure 4I). These findings confirm that Doppel loss in tumor endothelial cells induces transcriptomic reprogramming away from the tip cell phenotype, leading to impaired sprouting angiogenesis, reduced vessel density, and ultimately diminished tumor growth.

We also inoculated syngeneic EL4 thymoma (Figure S6A–C) into WT, Dpl^+/−^ and Dpl^−/−^ mice and CT26 colon cancer cells (Figure S6D–F) into WT and Dpl^+/−^ mice. In both models, Doppel deficient mice consistently exhibited reduced tumor growth and vessel formation compared to controls. Concordantly, Kaplan–Meier analyses of TCGA datasets showed that high Doppel (*PRND*) expression was associated with poorer survival in patients with thymoma, colorectal adenocarcinoma, and stomach adenocarcinoma (Figure S6G–I). These findings suggest that Doppel plays a central role in pathological angiogenesis, independent of tumor type. Interestingly, the suppression of tumor growth observed in Doppel‐deficient mice was partially rescued by intravenous administration of recombinant Doppel at 1 mg kg^−1^ once every 2 days in B16‐F10 melanoma and EL4 thymoma models (Figure S6J,K). Administration of fluorescence‐tagged rDpl showed co‐localization with CD31^+^ and VEGFR2^+^ blood vessels within the tumor tissue. This indicates that Doppel treatment can reverse the loss of endogenous Doppel in vivo, further confirming its role as a pro‐angiogenic mediator (Figure S6L). Importantly, Doppel deletion did not affect vessel formation in non‐pathological settings. Wound healing assays demonstrated that Doppel genotype had no impact on the rate of wound closure in mice (Figure S7A,B). Matrigel plug angiogenesis assays showed no significant differences in vascular density between WT and Doppel‐KO mice (Figure S7C,D), suggesting that Doppel specifically regulates pathological, but not physiological, angiogenesis.

### 2.7 Development of First‐in‐Class Anti‐Doppel Monoclonal Antibodies Targeting Conserved VEGFR2‐Binding Interfaces

**FIGURE 5:**
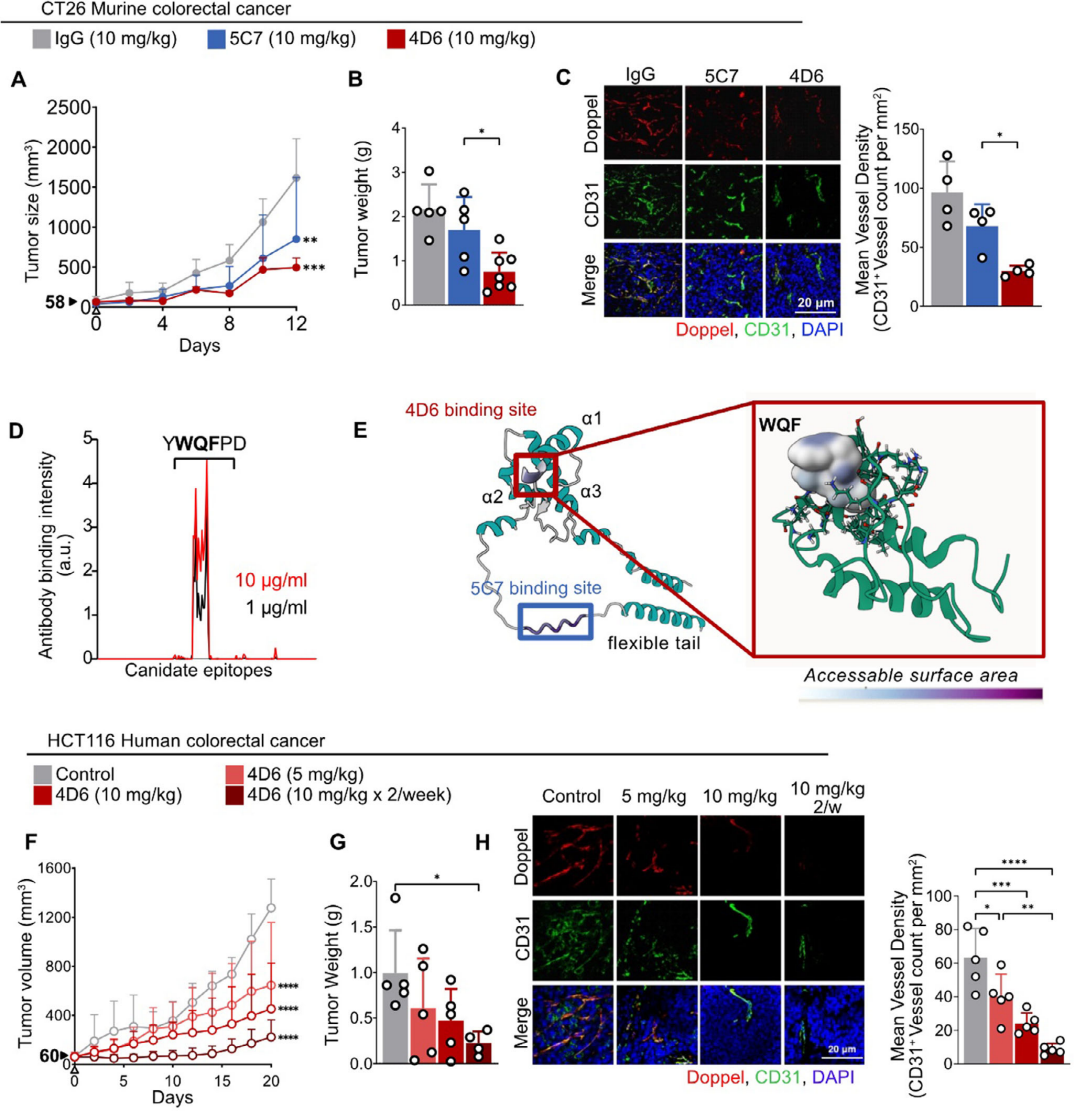
Doppel‐blocking antibodies reduce vascular density and suppress tumor growth. (A) Tumor growth curves of CT26 colorectal tumors treated with human IgG isotype control and anti‐Doppel antibodies: 5C7 (blue) and 4D6 (red) at 10 mg kg^−1^, *n* = 4–5. Two‐way ANOVA followed by Tukey's multiple‐comparison test; *p* ^**^< 0.01, *p* ^***^< 0.001 vs IgG. (B) Tumor weights at endpoint, pooled from two independent experiments. *p* ^*^< 0.05. (C) Representative immunofluorescence of CT26 tumor sections for staining with Doppel, red; CD31, green; DAPI, blue. Scale bar, 20 µm. Right panel: Quantification of mean vessel density based on CD31 staining. Data represents vessel counts per unit area. *p* ^*^< 0.05. (D) PEPperMAP epitope mapping showing the binding intensity of 4D6 at 10 µg mL^−1^ and 1 µg mL^−1^. The strongest signal corresponds to adjacent peptides containing the consensus motif YWQFPD; WQF represents the minimal core binding sequence. (E) Structural modeling of 4D6 and 5C7 epitope mapped on the AlphaFold‐predicted structure of Doppel, highlighting epitope accessibility and proximity to secondary structural elements. Inset: NMR‐resolved structure of human Doppel showing the core binding motif WQF. Accessible surface area was calculated and color coded using Mol^*^ visulaization. (F) Growth curves of HCT116 colorectal tumors treated with anti‐Doppel antibody (4D6) at indicated dosing regimens, *n* = 4–5. Two‐way ANOVA followed by Tukey's multiple‐comparison test; *p* ^****^< 0.0001 vs control. (G) Tumor weights at endpoint. *p* ^*^< 0.05. (H) Representative immunofluorescence images of tumors from each group stained for Doppel, green; CD31, red; DAPI, blue. Scale bar, 20 µm. Right panel: Quantification of mean vessel density per area. All data are presented as mean ± standard deviation (SD). Arrows indicate the treatment initation and tumor size at that time. Statistical analysis was performed using one/two‐way ANOVA followed by Tukey's multiple‐comparison test; *p* ^****^< 0.0001, *p* ^***^< 0.001, *p* ^**^< 0.01, *p* ^*^< 0.05, nonsignificant comparisons not shown.

To explore the therapeutic potential of targeting Doppel in tumor angiogenesis, we generated and screened multiple murine anti‐Doppel monoclonal antibodies (mAbs). Six candidates (7D9, 1B12, 1C8, 5C7, 4D6, and 6F11) were initially selected based on high binding affinity to recombinant murine Doppel (*PRND*) protein by ELISA (data not shown). Among these, 4D6 and 5C7 were prioritized for further study due to their ability to inhibit VEGF‐induced sprouting of endothelial spheroids. We next evaluated the in vivo efficacy of 4D6 and 5C7 in CT26 colorectal tumor models. Both antibodies reduced tumor growth, with 4D6 demonstrating potent activity with once‐a‐week dosing of 10 mg kg^−1^ leading to a 71.1% ± 4.2% reduction in tumor size. Once‐a‐week dosing of 10 mg kg^−1^ 5C7 also exhibited reduced tumor growth and size but the magnitude of inhibition was significantly lower than that achieved with 4D6. (Figure 5A,B; Figure S8A,B). Immunohistochemical staining also confirms that both 4D6 and 5C7‐treatment significantly reduced Doppel‐ and CD31‐positive vessels and overall vessel density compared to controls (Figure 5C; Figure S8C,D); however, 4D6 showed slightly higher effect than 5C7.

An explanation for the slight differences in efficacy can be found in the binding epitopes of the two antibodies. To characterize the binding specificity of 4D6, we performed PEPperMAP Epitope Mapping using 15‐amino‐acid linear peptides with a 14‐amino‐acid overlap. 4D6 exhibited a strong and specific monoclonal response against a single epitope‐like spot pattern, YWQFPD, with the WQF sequence identified as the minimal short core motif required for binding (Figure 5D). Structural analysis using the NMR‐resolved structure of human Doppel (PDB ID: 1LG4) and AlphaFold‐predicted murine Doppel models revealed that the YWQFPD motif resides in a surface‐accessible region at the end of the α1 domain of Doppel (Figure 5E; Figure S9A).

In contrast, 5C7 recognized a distinct epitope (IKHRFKWNRK) located outside the NMR resolved structure (Figure S9B). In the human Doppel AlphaFold model this motif mapped to a flexible region within the N‐terminal tail of the protein with low AlphaFold pLDDT confidence scores, implying reduced structural definition but higher accessibility (Figure 5E; Figure S9C). Sequence alignment of Doppel orthologs from 282 eukaryotic species confirmed the evolutionary conservation of both epitopes, although the 4D6‐binding motif exhibited greater conservation (Figure S9D). These findings suggest that the highly conserved globular α1 domain targeted by 4D6 represents a structurally and functionally critical region, likely accounting for its superior therapeutic efficacy compared to the more flexible N‐terminal epitope recognized by 5C7. Consistent with these structural observations, downstream signaling analysis in HUVECs demonstrated that treatment with anti‐Doppel antibodies led to reduced VEGFR2 pathway activation, confirming that Doppel blockade functionally suppresses angiogenic signaling (Figure S9E). We further evaluated the efficacy of α‐Doppel in HCT116 human colorectal cancer models in a dose dependent manner. 4D6 treatment dose dependently reduces tumor volume, weight, Doppel‐ and CD31‐positive vessels and overall vascular density compared to controls (Figure 5F–H; Figure S9F). Together, these results confirm robust ^Tip^ECs targeting and anti‐tumor efficacy of anti‐Doppel mAbs.

### 2.8 Blocking Doppel Expressing Tumor ^Tip^ECs Using 4D6 as an Effective Therapeutic Strategy

**FIGURE 6:**
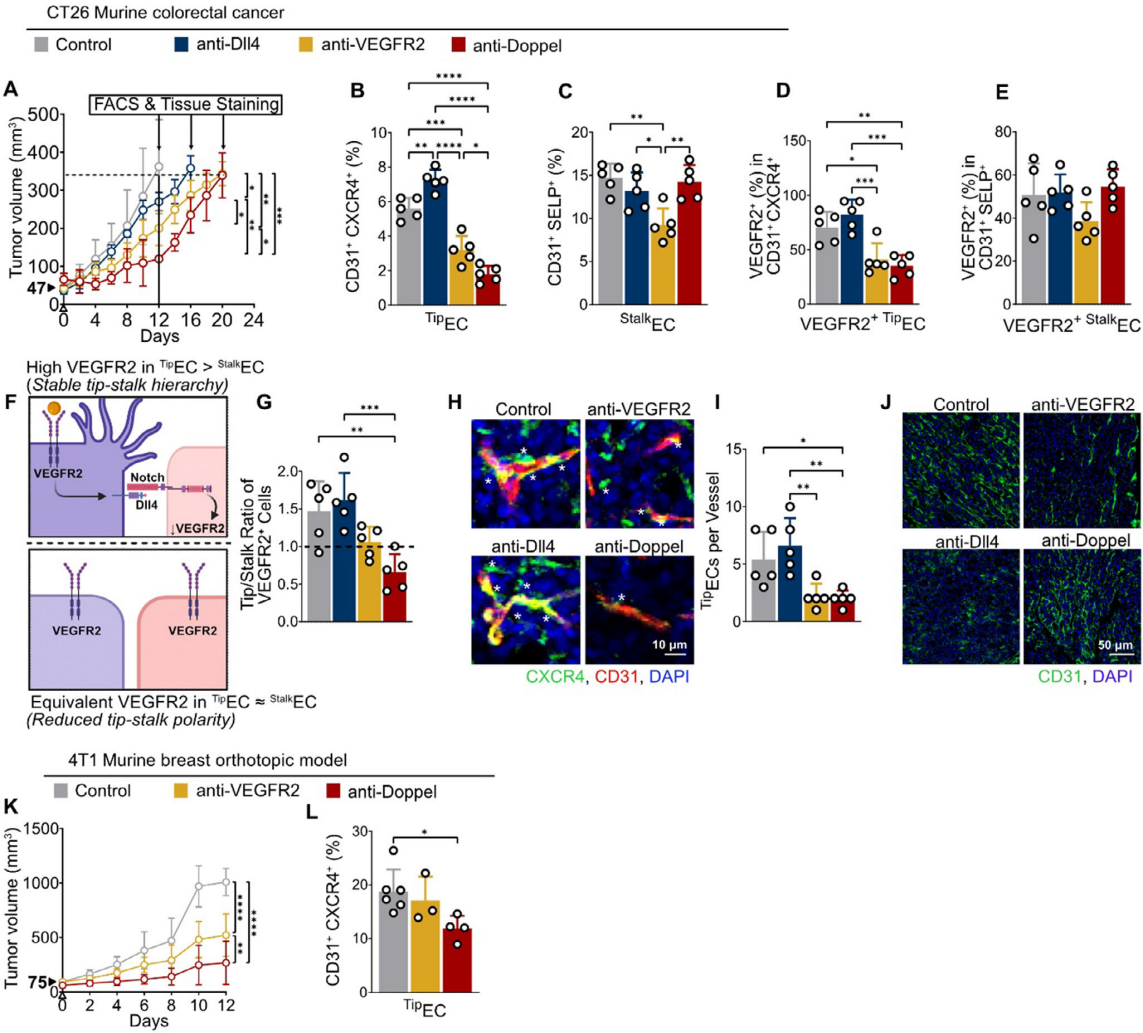
Selective depletion of ^Tip^ECs via Doppel blocking antibodies. (A) Tumor growth curves following treatment with anti‐Dll4 (navy), anti‐VEGFR2 (yellow), anti‐Doppel (red) antibodies, as well as control (gray). Mice were treated at 10 mg kg^−1^ once every 4 days, *n* = 7. Two‐way ANOVA followed by Tukey's multiple‐comparison test on day 12. (B,C) Flow cytometric quantification of endothelial subtypes in tumors: CD31^+^CXCR4^+^^Tip^ECs (B) and CD31^+^SELP^+^^stalk^ ECs (C). (D,E) Proportion of VEGFR2^+^ cells in ^Tip^EC (D) and ^stalk^EC (E) subsets. (F) Schematic representation of ^Tip^EC states based on relative VEGFR2 expression. Top: Highly differentiated ^Tip^ECs exhibiting a high tip/stalk VEGFR2^+^ ratio. Bottom: Competing or unstable ^Tip^ECs characterized by a low tip/stalk VEGFR2^+^ ratio, indicating phenotypic plasticity and competition with neighbouring ^Stalk^EC. (G) Quantification of VEGFR2^+^^Tip^EC/^Stalk^ EC ratio in tumors from each group. (H) Representative confocal images of tumor vasculature stained for endothelial and ^Tip^EC markers. White asterisks indicate CD31^+^CXCR4^+ Tip^ECs. CXCR4, green; CD31, red; DAPI, blue. Scale bar, 20 µm. (I) Quantification of ^Tip^ECs per vessel. Dots indicate individual vessels. (J) Representative immunofluorescence images of liver tissue sections of mice treated with anti‐VEGFR2 antibody, anti‐Dll4 antibody, and anti‐Doppel antibody, as well as control. red; CD31, green; DAPI, blue. (K) Tumor growth of 4T1 orthotopic tumors treated with control IgG (gray), anti‐Doppel antibody (red), and anti‐VEGFR2 (yellow). Mice were treated at 10 mg kg^−1^ once every 4 days, *n* =7. Two‐way ANOVA followed by Tukey's multiple‐comparison test. (L) Flow‐cytometric quantification of tip endothelial cells (^Tip^EC: CD31^+^CXCR4^+^). All data are presented as mean ± standard deviation (SD). Arrows indicate the treatment initiation and tumor size at that time. Statistical analysis was performed using one/two‐way ANOVA followed by Tukey's multiple‐comparison test unless otherwise stated; *p* ^****^< 0.0001, *p* ^***^< 0.001, *p* ^**^< 0.01, *p* ^*^< 0.05, nonsignificant comparisons not shown.

Finally, we compared different antibodies known to target ^Tip^EC with anti‐Doppel antibodies to show that selective depletion of ^Tip^ECs is sufficient to reduce tumor growth. Thus, we treated tumor‐bearing mice with three antibodies against: α‐VEGFR2 (ubiquitous EC expression), α‐Dll4 (regulates tip‐stalk ECs dynamic), α‐Doppel (selective to ^Tip^ECs) at the same dose and frequency. We collected the tumors at ∼350 mm^3^ for all the groups and analyzed tumor ^Tip^EC and ^Stalk^EC fractions. All antibodies, α‐VEGFR2, α‐Dll4 and α‐Doppel, showed significant delays in tumor growth (Figure 6A). We observed that α‐Doppel was effective in decreasing the number of ^Tip^ECs without affecting the ^stalk^ECs number (Figure 6B,C). In contrast, α‐VEGFR2 decreased both ^stalk^ECs and ^Tip^ECs, while α‐Dll4 increased ^Tip^ECs without affecting ^stalk^ ECs, consistent with previous reports [31, 35, 36]. We further evaluated VEGFR2 expression levels within endothelial subpopulations. We found that α‐Doppel reduced the percentages of VEGFR2^+ Tip^ECs but not VEGFR2^+ stalk^ECs, whereas α‐VEGFR2 decreased VEGFR2^+^ in both populations (Figure 6D,E).

Because VEGFR2 expression reflects tip/stalk competition dynamics, we next calculated the ratio of VEGFR2^+^^Tip^ECs to VEGFR2^+^^Stalk^ECs as a surrogate for tip cell dominance. In untreated tumors, ^Tip^EC naturally predominate (ratio = 1.46), reflecting the pro‐angiogenic tumor microenvironment (Figure 6F,G). Anti‐Dll4 treatment, by blocking Notch‐mediated lateral inhibition, further elevated the tip/stalk ratio to 1.60. However, such unchecked ^Tip^EC expansion without ^Stalk^EC support is known to result in unstable, non‐functional vasculature [35]. Anti‐VEGFR2, by blocking sprouting initiation, reduced both ^Tip^ECs and ^Stalk^ECs, decreasing the tip/stalk ratio to 1.06. Strikingly, anti‐Doppel treatment uniquely suppressed VEGFR2^+^^Tip^ECs while sparing ^stalk^ECs, shifting the balance in favor of ^stalk^EC (ratio = 0.66). Given that Doppel promotes Dll4 expression and stabilizes ^Tip^EC identity, these findings suggest that Doppel blockade disrupts VEGFR2‐driven dominance in ^Tip^EC, thereby suppressing ^Tip^EC selection and maintenance. Immunofluorescent images confirmed the findings of the flow cytometric analysis, showing a marked decrease in ^Tip^EC per vessel, identified with CXCR4 and CD31 staining respectively, after anti‐Doppel and anti‐VEGFR2 treatment. (Figure 6H,I). 4D6 treatment had no detectable adverse effects on body weight, histopathology, or blood chemistry (data not shown) as well as no changes in liver vessel density which was evident for both α ‐VEGFR2 and α‐Dll4 (Figure 6J), suggesting that Doppel blockade selectively targets tumor ^Tip^ECs without systemic toxicity.

To evaluate the in vivo efficacy of Doppel‐targeting therapy in a highly angiogenic, treatment‐refractory setting, we employed the 4T1 murine orthotopic breast cancer model. Mice were treated with either a murine‐cross‐reactive ramucirumab analogue anti‐VEGFR2 antibody (DC101) or an anti‐Doppel monoclonal antibody (Figure 6K). Both anti‐VEGFR2 and anti‐Doppel treatment resulted in tumor volumes significantly lower than control. Doppel inhibition was more effective than VEGFR2 inhibition, confirming that Doppel blockade is a viable therapeutic strategy. We next quantified the CD31^+^CXCR4^+^ endothelial subset to determine whether Doppel blockade selectively perturbs the ^Tip^EC population (Figure 6L). Flow cytometry revealed that control tumors contained a robust population of ^Tip^ECs, representing ∼20%–25% of total CD31^+^ cells. Anti‐Doppel treatment significantly decreased the frequency of ^Tip^ECs to ∼12%–15% (*p* < 0.05), indicating selective depletion of the angiogenic endothelium that drives vessel sprouting.

## 3 Discussion

Recent advances in single‐cell transcriptomics and spatial profiling have enabled more nuanced classification of endothelial subtypes, such as tip and stalk cells, based on their specialized functions [3, 37, 38]. Targeting these specialized subpopulations offers a more precise approach to modulate pathological angiogenesis while sparing vessels critical for normal vascular homeostasis. In this study, we identify Doppel as a key regulator of ^Tip^EC function, particularly within the context of tumor angiogenesis. By analyzing publicly available single‐cell RNA sequencing datasets alongside our own bioengineered in vitro and genetic models, we show that Doppel marks a highly specific subpopulation of ^Tip^ECs and promotes both their selection and functional activation. Mechanistically, Doppel acts as a cofactor for the VEGFR2 receptor, enhancing its phosphorylation even at sub‐threshold levels of VEGF. This amplifies downstream signaling through kinases such as Src, ERK1/2, and c‐Abl, which are integral to cytoskeletal remodeling and cell motility [22]. Further, Doppel appears to regulate tip‐stalk dynamics through the VEGFR2–Dll4–Notch axis. In canonical angiogenesis, VEGF‐induced Dll4 expression in ^Tip^EC activates Notch signaling in adjacent cells, suppressing VEGFR2 and promoting stalk cell identity [30, 31]. This lateral inhibition preserves the tip‐stalk balance but remains plastic [31]. Our findings suggest that Doppel aberrantly modulates the VEGFR2–Dll4–Notch axis, disrupting the balance between tip and stalk cell formation during sprouting angiogenesis. By hyperactivating VEGFR2 at the leading edge of ^Tip^ECs and inducing elevated Dll4 expression, Doppel appears to reduce ^Tip^EC plasticity and interfere with the canonical Dll4‐Notch axis that regulates stalk cell identity in adjacent ECs. However, further mechanistic studies are needed to clarify Doppel's precise role in modulating Dll4–Notch signaling during ^Tip^EC induction.

A defining feature of Doppel is its highly restricted expression pattern. Doppel is virtually absent from normal adult tissues, except in the testis where it supports sperm maturation. Unlike canonical pro‐angiogenic genes such as VEGF or Dll4, Doppel is largely absent from healthy adult vasculature and appears to be selectively re‐expressed during pathological angiogenesis. Notably, Doppel knockout mice develop normally without congenital defects aside from male infertility, in contrast to the embryonic lethality or vascular abnormalities observed with VEGF or Dll4 deficiency [32, 33, 39, 40]. Although Doppel has been linked to neonatal brain vasculature development, it does not seem to be critical for this process, nor for other physiological angiogenic events such as the wound healing [17]. However, tumor‐induced angiogenesis is markedly impaired in Doppel‐deficient mice. Doppel expression has also been reported in other pathological angiogenic settings, including hereditary hemorrhagic telangiectasia and pulmonary arterial hypertension, further supporting its context‐specific role in driving aberrant vascular growth [10, 41].

Angiogenesis begins with the selection of ^Tip^ECs, which migrate toward the angiogenic stimulus, guiding trailing ^Stalk^ECs [1, 42]. ^Tip^ECs actively survey the tumor microenvironment for angiogenic cues and recognize each other to connect sprouts. Without ^Tip^ECs, ^Stalk^ECs lose their directional cues for lumen formation. Thus, targeting ^Tip^ECs offers a more selective approach than conventional therapies that indiscriminately target all angiogenic endothelial populations; pathological and physiological, tumor‐associated and normal, tip and stalk alike; such as anti‐VEGF/VEGFR2 that fails to account for the dynamic and heterogenous nature of the endothelium [29, 42, 43, 44, 45]. VEGF/VEGFR2 blockade shuts down angiogenic sprouting altogether, resulting in a reduction of total ECs, sometimes leading to tumor inhibition at the cost of life‐threatening side effects due to its effect on healthy vessels, such as severe hemorrhaging [46]. On the other hand, targeting other ^Tip^ECs markers, e.g. Dll4, disrupts lateral inhibition and releases ^Stalk^ECs from Notch‐mediated suppression, resulting in an uncontrolled expansion of ^Tip^ECs and generating chaotic, nonfunctional sprouting. In contrast, selective disruption of Doppel‐positive ^Tip^ECs stops sprout initiation and restrains aberrant expansion of ^Tip^EC. Consequently, ^Tip^ECs cannot migrate toward the stimulus and formation of vessels is highly affected. Notably, Doppel inhibition achieved a degree of tumor suppression comparable to VEGFR2 inhibition, underscoring that selective regulation of the tip compartment alone is sufficient to curb tumor growth.

While the VEGF/VEGFR2 signaling axis is critical for EC survival and vessel maintenance, and its inhibition represents a well‐established method of inducing broad vascular regression, our data indicates that selective restriction of the tip compartment alone can achieve comparable antitumor effects. Because rapidly growing tumors cannot depend solely on pre‐existing vessels to meet metabolic demands, suppression of ^Tip^ECs‐mediated sprouting constitutes a biologically sound mechanism for limiting tumor progression [42]. Consistent with this, our data show that selective blockade of Doppel not only restricts vascular expansion but also reduces microvessel density and perfused vessel area in established tumors, demonstrating a meaningful impact on tumor vasculature. This targeted strategy may avoid the systemic toxicities associated with pan‐endothelial VEGF inhibition while maintaining antitumor efficacy, supporting anti‐Doppel therapy as a complementary and mechanistically distinct approach to angiogenesis targeting. This positions Doppel as an unique and selective node within the angiogenic signaling network, offering a promising targeting for pathological angiogenesis while sparing normal vascular function [47].

Consistent with this specificity, anti‐Doppel antibody treatment in our study did not perturb normal vasculature, including in the liver (Figure 6H), in contrast to the broad vascular effects often observed with conventional anti‐angiogenic agents. Off‐target effects in the brain or testis, the only two normal organs where Doppel is known to be expressed, are also unlikely, as both organs are protected by specialized blood–tissue barriers that limit antibody penetration [47, 48]. Together, these observations support the therapeutic feasibility of Doppel inhibition with a low risk of adverse effects in non‐target tissues. In this context, our findings position Doppel as a molecular “gatekeeper” that is reactivated under pathological conditions to promote aberrant vessel formation. Targeting these pathological Doppel‐high ^Tip^ECs offers a unique opportunity to inhibit disease‐associated angiogenesis selectively, while preserving normal vascular function—a major unmet goal in anti‐angiogenic therapy [29].

## 4 Experimental Section/Methods

### 4.1 Single Cell Data Processing and Visualization

Processed single‐cell RNA‐sequencing data of endothelial cells and associated metadata were downloaded from the original publication [4]. Data were analyzed using the Seurat package in R. Cell identities were annotated using the provided metadata, and violin and density plots were generated to visualize expression patterns across cell types. Endothelial cells were then subset and stratified by cell type or *PRND* expression for downstream analysis. Differential expression analysis was performed using FindMarkers with Wilcoxon rank‐sum testing. Genes with adjusted *p* < 0.05 and |log_2_FC| > 0.25 were considered significant and used for pathway enrichment analysis via clusterProfiler, including Hallmark, GO, and KEGG pathways. Visualization of DEG results included volcano plots (ggplot2) and heatmaps (ComplexHeatmap) of the top 20 DEGs, with Z‐score–scaled expression across *PRND*‐positive and ‐negative ^Tip^ECs.

### 4.2 Generation of Doppel Knockout (KO) Mice

All animal experiments and surgical procedures were performed according to the regulations of the Institutional Animal Ethics Committee of the Seoul National University, Seoul, animal care facility, as described in the Regulation for the Care of Animals (IACUC # SNU‐070822‐5). We created a complete Doppel KO mouse model from immunogenic C57BL/6 mice [49]. Cryopreserved embryos (EM:02571), from C57BL/6;129P2‐Prnd^tm1Aag/Cnrm^ mice, were purchased from European Mouse Mutant Archive (EMMA, Italy). Doppel mutant embryos were transferred into the oviduct of half‐day pseudo‐pregnant ICR female mice and bred at Macrogen Inc. (Seoul, South Korea). For genotyping, genomic DNA was extracted from the tails of pups. Heterozygous mice carrying the mutated *PRND* allele were backcrossed and bred further for >5 generations to produce both heterozygous (Dpl+/‐) and homozygous (Dpl‐/‐) Doppel KO mice. We used male and female Doppel wild‐type (WT) and KO littermates in our tumorigenesis studies.

### 4.3 Tumor Transplantation, Wound Healing and Matrigel Plug Assays in Doppel KO Mice

Syngeneic B16F10 melanoma (2 x 10^6^ cells per mouse), EL4 thymoma (1 x 10^6^ cells per mouse), and CT26 colon (5 x 10^6^ cells per mouse) cancer cell lines were inoculated subcutaneously in WT, Dpl+/‐, and Dpl‐/‐ KO male and female littermates (*n* = 3∼4). Tumors were measured with a caliper for 3 weeks after inoculation and tumor volumes were calculated using the formula: volume = length x width^2^ x 0.52. We created a wound in the opposite flank of tumor‐bearing Doppel WT and KO mice by using a Uni‐Punch Biopsy Punch (Premier Medical, Medline, IL), and measured the wound with a digital caliper once a day for 6 days. On day 6, skins were removed for immunofluorescence staining of blood vessels in the wounded area. The CD31 positive surface area in the region of interest (ROI, granulation area) was averaged from 5 wounds per group (4 sections per wound), and reported as a percent of the ROI area. For angiogenesis assays, WT, Dpl+/‐, and Dpl‐/‐ KO mice were injected subcutaneously with 500 µL of Matrigel (BD Biosciences) containing 300 ng of mouse VEGF and mouse bFGF (Peprotech). After 6 days of implantation, the Matrigel plugs were removed. Part of the plugs were homogenized, and hemoglobin content was measured. The plugs were also embedded in paraffin, sectioned, and stained with anti‐CD31 antibody to assess the microvessel density.

### 4.4 Murine Doppel Protein Expression and Purification

To obtain the recombinant murine Doppel protein (murine rDpl), murine *PRND* (*mPRND*) gene (pCMV6‐mPrnd, Origene) was prepared through PCR amplification using appropriate primers, encoding NH2‐NdeI‐mPrnd‐XhoI‐COOH so that it can express His‐tagged protein at the C‐terminus. After ligation into a pET‐21a plasmid vector to construct the expression vector pET‐21a‐mPrnd, the construct vector was transformed into E. coli (BL21(DE3)RIPL[F‐ompThsdS(rB‐mB‐)]) with ampicillin‐resistant selection. Transformed cells with the above construct were grown at 37°C to an OD600 of 0.6 in LB medium containing ampicillin and protein expression was induced by 1 mm IPTG at 37°C for 4 h. After induction, cells were harvested by centrifugation, and the pellets were suspended in lysis buffer (50mm NaH2PO4, 300 mm NaCl, 8m Urea, 5mm Imidazole, pH 8.0) and homogenized with an ultrasonic processor. Recombinant murine rDpl, with a size of 18.6 kDa, was purified by a Ni‐NTA chromatography step under denatured conditions. A total of 15.6 mg purified protein was stored at a concentration of 5.2 mg mL^−1^ in lysis buffer that contained 153mm Imidazole. Murine rDpl protein was analyzed by SDS‐PAGE and western blotting using commercially available anti‐Doppel polyclonal antibody.

### 4.5 Synthesis of rDpl‐cy5.5

A 4.4 mg mL^−1^ stock of human or murine rDpl was diluted 4‐fold in bicarbonate buffer (pH 8.5) and reacted with Cy5.5‐NHS at a 1:5 molar ratio. After 2 h in room temperature, the excess dye was removed with a PD‐10 column. As a control, the same mixture was prepared without rDpl, and the same fractions were collected from the PD‐10 column.

### 4.6 Generation of Anti‐Doppel Monoclonal Antibodies

To create Doppel‐mAb, mice were immunized with murine rDpl along with pristane (a shark oil‐derived saturated terpenoid alkane, Sigma) as an immunological adjuvant. B‐cells were collected from mice and tested for anti‐mouse Doppel titers using an ELISA. B‐cells with the highest titer were fused with myeloma cells [50, 51]. The resulting hybridoma cells with the highest antibody titer were expanded, centrifuged to collect supernatants, screened by ELISA for binding with mouse Doppel, and tested for their ability to detect Doppel protein in TEC lysates. Six independent clones (7D9, 1B12, 1C8, 5C7, 4D6, and 6F11) were identified, parent clones were subcloned multiple times, and the final subclones were assayed and scaled up. Clones were purified by protein A affinity chromatography.

### 4.7 PEPperMAP Epitope Mapping

The C‐ and N‐termini of Doppel antigen were elongated by neutral GSGSGSG linkers to avoid truncated peptides. The elongated antigen sequence was translated into 15 amino acid peptides with a peptide‐peptide overlap of 14 amino acids. The resulting peptide microarrays contained 179 different peptides printed in duplicate (358 peptide spots) and were framed by additional HA control peptides (YPYDVPDYAG, 74 spots). Pre‐staining of one of the peptide microarray copies was done with the secondary goat anti‐mouse IgG (H+L) DyLight680 antibody (1:5000) in incubation buffer to investigate background interactions with the antigen‐derived peptides that may interfere with the main assays. Subsequent incubation of other peptide microarray copies with mAb 4D6 and 5C7 at concentrations of 1 and 10 µg mL^−1^ in incubation buffer was followed by staining with the secondary antibody and read‐out at a scanning intensity of 7 (red). The additional HA peptides framing the peptide arrays were subsequently stained with control antibody mouse monoclonal anti‐HA (12CA5) DyLight800 as internal quality control to confirm the assay quality and the peptide microarray integrity.

Quantification of spot intensities and peptide annotation were based on the 16‐bit gray scale tiff files at a scanning intensity of 7 that exhibit a higher dynamic range than the 24‐bit colorized tiff files; microarray image analysis was done with PepSlide Analyzer. A software algorithm breaks down fluorescence intensities of each spot into raw, foreground and background signal, and calculates averaged median foreground intensities and spot‐to‐spot deviations of spot duplicates. Based on averaged median foreground intensities, an intensity map was generated and interactions in the peptide map highlighted by an intensity color code with red for high and white for low spot intensities. We further plotted averaged spot intensities of the assays with the antibody samples against the antigen sequence from N‐ to C‐terminus to visualize overall spot intensities and signal‐to‐noise ratios. The intensity plots were correlated with peptide and intensity maps as well as with visual inspection of the microarray scans to identify the epitopes of the antibody samples.

### 4.8 Antibody Epitope Visualization

PDB files of the AlphaFold‐predicted structures of human and mouse Doppel were downloaded from the AlphaFold Protein Structure Database under accession numbers AF‐Q9UKY0‐F1‐v4 and AF‐Q9QUG3‐F1‐v4 respectively. This as well as the NMR‐resolved structure of human Doppel (PBD: 1LG4) were visualized using Mol^*^ [52]. Accessible surface areas and core binding motifs were computed and color‐coded within Mol^*^ to aid in structural comparison and epitope mapping.

### 4.9 Sequence Conservation Analysis

Doppel protein sequences from 282 eukaryotic species were retrieved from OrthoDB (ortholog group 9523143at2759). Multiple sequence alignment was performed using Clustal Omega [53], and the resulting alignment was visualized using WebLogo3 [54] to highlight conserved residues across species.

### 4.10 Tumor Treatments

CT26 mouse (5 x 10^6^ cells) and HCT116 human (1 x 10^7^ cells) colon cancer cells were subcutaneously inoculated at the dorsal flank of C3H/HeN mice (male, 6–7 weeks old, *n* = 5) and Balb/c nude mice (male, 6–8 weeks old, *n* = 5–7), respectively. When tumors reached 50–70 mm^3^, mice were treated via tail vein injections with 5C7 and 4D6 anti‐Doppel mAbs (5 and 10 mg kg^−1^) once per week. For another group of mice, 4D6 anti‐Doppel mAb was administered intravenously twice per week at dose of 10 mg kg^−1^. For a separate study, mice received tail‐vein injections of anti‐VEGFR2 mAb (murine‐cross‐reactive ramucirumab analogue, clone DC101), anti‐Dll4 mAb (clone HMD4‐2), or anti‐Doppel mAb at 5 or 10 mg kg^−^^1^ once every 4 days. For the orthotopic model, 4T1 murine breast cancer cells (5 × 10^6^ cells) were inoculated into the fourth mammary fat pad. Treatment began when tumors reached 50–70 mm^3^. Tumors were measured with a caliper at regular intervals and tumor volumes were calculated using the formula: volume = length x width^2^ x 0.52. 4 of the 7 mice treated with anti‐VEGFR2 died at 10 days after the initiation of treatment. Mice were sacrificed when CT26 tumors reached a volume greater than 2.5 cm^3^ and HCT116 tumors reached a volume greater than 1.2 cm^3^. Orthotopic models were sacrificed 12 days after initiation of treatment for analysis.

### 4.11 Tumor Cell Culture

SCC7 (RRID: CVCL_V412), CT26 (RRID: CVCL_7254), B16F10 (RRID: CVCL_0159), EL4 (RRID: CVCL_0255), HCT116 (RRID: CVCL_0291) cell lines were purchased from the American Type Culture Collection (ATCC). Cells were maintained in the following media: SCC7 and CT26 in RPMI‐1640, B16F10 and EL4 in DMEM, and HCT116 in McCoy's 5A medium, each supplemented with 10% fetal bovine serum, 100 U mL^−1^ penicillin and 100 µg mL^−1^ streptomycin. All cells were cultured at 37°C in a humidified incubator with 5% CO_2_ and were tested routinely for mycoplasma contamination.

### 4.12 Whole Mount, Immunofluorescence, and Immunohistochemical Staining of Mouse Tumor Tissues

Mouse tissue sections (skin, heart, and tumors) and Matrigel plugs were embedded into paraffin (5 µm thickness). Paraffin sections were deparaffinized, incubated with proteinase K, heated at 95°C for 20 min in citrate buffer (pH 6.0, Invitrogen), and treated with peroxidase blocking reagent (Dako). For immunohistochemical staining, sections were incubated with antibody against anti‐mouse CD31 (1:100, clone MEC13.3, 550274, BD Biosciences) followed by an HRP‐conjugated secondary antibody (Dako) and visualized by DAB (diaminobenzidine) staining. Sections were lightly counterstained with hematoxylin. For whole mount staining, tumors were dissected, cut into pieces (∼2 x 2 mm), and fixed in methanol containing 25% DMSO for 24 h at 4°C. Next day, the tumor pieces were washed three times with sterile PBS for 1.5 h, blocked for 3 h with 5% BSA, and finally incubated with the primary antibody overnight at 4°C under gentle agitation. As a control, tumor pieces were incubated with control IgG instead of the primary antibodies. After overnight incubation, the tumor pieces were washed three times with TBST for 3 h at 4°C under gentle agitation and stained overnight with fluorescence‐labeled secondary antibodies at 4°C under gentle agitation. Finally, the tumor pieces were counterstained with Hoechst dye, embedded in mounting medium, and sealed the slides with Pertex so that they cannot dry out. The structure of the tumor vessels was analyzed by confocal microscopy. For Doppel and CD31 detection, tissue sections were stained with custom‐made Rabbit anti‐mouse Doppel polyclonal antibody (Gift from Dr. Joseph McCarty) followed by Alexa 488‐linked donkey anti‐rabbit (1:200, A11055, Invitrogen) and PE‐linked rat anti‐mouse CD31 antibody (1:200, 553373, BD Biosciences), respectively. For VEGFR2 staining, goat anti‐mouse VEGFR2 polyclonal antibody (1:200, AF644, R&D Systems) followed by Alexa 488‐linked donkey anti‐goat antibodies were used. As a control, few tumor pieces were incubated with control goat IgG, followed by Alexa 488‐linked donkey anti‐goat and PE‐linked rat anti‐mouse CD31 antibodies.

### 4.13 Tumoral Endothelial Cell (TEC) Isolation and Analysis

Tumoral endothelial cells were isolated using double marker and followed by a procedure published earlier with some modifications [11]. Briefly, tumors were grown subcutaneously in the flank of C_3_H/HeN mice and resected, minced using two surgical blades, and digested in 9 mL collagenase and 1 mL dispase solution per gram of tissue. Tissues were incubated for 30 min in a 37°C water bath, under continuous agitation. Subsequently, 75 µL DNaseI solution per 10 mL cell suspension was added and incubated for another 30 min at 37°C with continuous agitation. Digested tissues were sieved through a 100‐µm cell strainer and single cells were separated. Cells were collected by centrifugation at 400 *g* for 7 min at room temperature. To remove red blood cells, granulocytes, nonvital cells and cell debris, cells were resuspended in 10 mL Ficoll separation medium (per gram of starting material) and carefully layer the suspension on 7.5 mL Ficoll‐Paque (pre‐warmed to room temperature). The interphase‐containing viable cells were transferred into a fresh tube. Cells were collected into FACS tubes and incubated with anti‐mouse CD31‐PE and anti‐mouse CD34‐FITC antibodies (at a final concentration of 2 µg mL^−1^). FACS machine was prepared by adjusting conditions with a sheath fluid pressure of 29.9 psi, a sorting frequency of 44 kHz and a plate voltage of 3500 V. Collected cells were suspended in 10 mL ECGM containing 10% FBS and centrifuged. Then finally, cells were plated in 0.2% gelatin‐coated 100 mm dishes and cultured overnight in TEC growth media that is supplemented ECGM containing 10% FBS and 10% cancer cells‐derived conditioned media. Next day, the media was replaced with fresh TEC growth media. Cells were grown for 2–3 days until confluent. Cell cultures were tested routinely for mycoplasma contamination. Cell morphology and growth characteristics were monitored regularly. For analysis of TECs, tumors were resected once they reached an equivalent size of approximately 300 mm^3^ and processed as previously described. The cells were then stained with CD31‐PE, CD45‐PerCP/Cy5.5, CXCR4‐Cy7, SELP‐APC, and VEGFR2‐AF549 and analyzed with a spectral flow cytometer.

### 4.14 Endothelial Cell Culture

Human dermal microvascular endothelial cells (HDMEC, Promo Cell, catalog number: C‐12212) and human umbilical vein endothelial cells (HUVEC, RRID: CVCL_0F27) were obtained from the commercial vendor (Promo Cell). HDMEC is a non‐immortalized, donor‐derived primary endothelial cell population and, as such, does not have a research resource identifier (RRID). HDMEC, HUVEC, and transfected cells were maintained in endothelial cell growth medium MV2 (ECGM, Promo Cell) supplemented with 100 U mL^−1^ penicillin and 100 µg mL^−1^ streptomycin according to the manufacturer's instructions. TECs were isolated from SCC7 and CT26 cancer cells as described previously. TECs were cultured in supplemented ECGM containing 10% FBS and 10% conditioned media in gelatin‐coated dishes. Condition media was prepared by incubating serum free media for 24 h in 80%∼90% confluent dishes of cancer cells (SCC7 or CT26) after which the supernatant was collected, centrifuged, and filtered through 0.2 mm syringe filters (Corning). The resulting CM were stored in aliquots at −80°C. For all experiments, TECs were used between passage numbers (P) 1 to 5 with population doublings up to 15.

### 4.15 Photolithographic Fabrication of VEGF‐Patterned Polyacrylamide Hydrogels

Photolithography was used to create polyacrylamide (PA) hydrogels with alternating VEGF‐containing (V^+^) and VEGF‐free (V^−^) stripes. A stripe pattern was designed in AutoCAD (Autodesk), consisting of 200 µm opaque (black) and 100 µm transparent lines. This pattern was printed onto a chrome‐coated quartz mask using high‐resolution printing (Advance Reproductions Corporation, MA). To fabricate the patterned hydrogels, a 5% PA solution without VEGF (V^−^) was first photo‐polymerized on a methacrylate‐treated 18 mm glass coverslip under UV light (365 nm) for 5 min, using 5% IrgaCure as the photo‐initiator. Next, a second 5% PA solution containing 50 µg mL^−1^ VEGF (V^+^) was layered on top. The photomask was positioned between the UV source and the hydrogel to allow selective crosslinking of the V^+^ solution in 100 µm stripes by blocking light over the 200 µm areas. This resulted in polymerized V^+^ stripes alternating with unpolymerized regions. Unpolymerized stripes were removed by washing with PBS, yielding hydrogels with interleaving V^+^ and V^−^ regions. Multiple gels were fabricated simultaneously from the same polymer batch to minimize variability. Hydrogels were then activated with sulfo‐SANPAH (1 mg mL^−1^) and functionalized with collagen (40 µg mL^−1^) and fibronectin (10 µg mL^−1^) in PBS for 4 h at 37°C.

### 4.16 Endothelial Cell Sprouting Assay

EC spheroids were prepared as described in the literature [55]. In brief, ECs were grown in gelatin‐coated dishes, trypsinized, and suspended in a mixture containing 80% growth media and 20% Methocel solution. Methocel was prepared by dissolving 6 g of methylcellulose (Sigma) in 500 mL of endothelial cell basal media (PromoCell). ECs were cultured in hanging drops of 25 µL such that 1000 ECs aggregate as spheroids; HUVEC, HDMEC, and Doppel transfected cells formed spheroids within 24 h of culture whereas TECs were cultured for 72 h to form spheroids. The generated spheroids were harvested and suspended in a solution that containing methocel with 20% FBS. Subsequently, the ice‐cold collagen solution (rat tail type I in 0.1% acidic acid) was mixed with 10% MEM 199 (10X, GIBCO; Invitrogen), followed by adding 10% 0.2 N NaOH to adjust the pH to 7.4. TEC spheroids were suspended in a 1:1 mixture of Methocel and neutralized collagen solution, along with 7D9, 1B12, 1C8, 5C7, 4D6, and 6F11 anti‐Doppel mAb clones at a concentration of 10 µg mL^−1^, in the presence or absence of mouse VEGF‐A164 (50 ng mL^−1^, Peprotech). The mixed solution (0.9 mL), containing 50 TEC spheroids, were pipetted into individual wells of a 24‐well plate and allowed to polymerize for 30 min at 37°C. In experiments with Doppel cDNA or mock cDNA transfected HDMEC cells, spheroids were also prepared and seeded in the presence or absence of VEGF‐A165 (50 ng mL^−1^, Peprotech). In another set of experiments, HUVEC and Hu^Dpl^ cell spheroids were seeded in the presence or absence of various concentrations of soluble Fc‐tagged recombinant human Doppel protein (human rDpl, a molecular weight of 55 kDa, ANRT, Korea) derived from HEK293F cells, which were further stimulated with 50 ng mL^−1^ of VEGF‐A165. VEGF activates ECs, thereby inducing the formation of tube‐like EC structures (sprouting EC). The plates were incubated at 37°C in 5% CO_2_ for 24 h. Sprouting EC spheroids were photographed, and sprouting was quantified by counting the cumulative sprout branch points per spheroid using light microscope (Nikon Ltd., Japan).

### 4.17 Cellular Studies

For the treatment of TECs, cells were starved in endothelial basal medium (EBM) containing 0.5% FBS overnight. The next day, cells were treated with different concentrations of anti‐doppel mAb clones for 30 min, followed by treatment of 100 ng mL^−1^ of mVEGF for additional 5–7 min. To analyze the phosphorylation of VEGFR2, serum‐starved HUVEC and Hu^Dpl^ cells were incubated different concentrations of VEGF‐A165 for 5 min. Phosphorylation of VEGFR2 was also compared with Doppel cDNA and mock cDNA transfected HDMEC cells. After 24 h of transfection, cells were serum deprived for 6 h and stimulated with 100 ng mL^−1^ of VEGF‐A165 for 30 min. To observe the interaction between Doppel and VEGFR2 in Doppel cDNA transfected or human rDpl‐treated HDMEC cells, lysates were immunoprecipitated with anti‐VEGFR2 antibody (55B11, Cell Signaling) followed by immunoblotting for VEGFR2 and Doppel. For VEGFR2 phosphorylation studies, serum‐starved HDMECs were pretreated with 1 µg mL^−1^ of human rDpl for 1 h followed by stimulation with or without 0‐200 ng mL^−1^ of VEGF‐A165 for 5 min. Total protein lysates were generated as described before. All experiments were performed in triplicates.

### 4.18 Immunoblotting

Proteins were extracted from lung, tumor tissues, or cells using lysis buffer as described earlier. Briefly, equal amounts of protein were resolved in 7.5%–12% SDS‐PAGE and blotted onto PVDF membranes (IPVH00010, Millipore). Blocking was performed in 5% BSA for 1 h and membranes were incubated in primary antibodies overnight at 4°C. Membranes were incubated with HRP­conjugated secondary antibody for 1 h. The following antibodies were used: Phospho‐VEGFR2 polyclonal antibody (1:2000, AF1766, R&D Systems), anti‐mouse VEGFR2 polyclonal antibody (1:1000, AF644, R&D Systems), anti‐human VEGFR2 monoclonal antibody (1:2000, 55B11, Cell Signaling), anti‐human VEGFR2 polyclonal antibody (1:2000, SAB4501645, Sigma–Aldrich), custom‐made mouse anti‐human Doppel monoclonal antibody (2B6), anti‐Pan‐cadherin monoclonal antibody (1:2000, C1821, Sigma), anti‐RPS20 antibody (1:1000, HPA003570, Sigma), GAPDH antibody (1:4000, MAB5718, R&D Systems), β‐actin antibody (1:4000, CC10028, Cell Applications) and horseradish peroxidase (HRP)‐conjugated secondary antibody to goat (1:2000, HAF109, R&D Systems), mouse (1:2000, HAF007, R&D Systems), rabbit (1:2000, HAF008, R&D Systems), goat TrueBlot (18‐8814, 1:2000, eBioscience), rabbit TrueBlot (18‐8816, 1:2000, eBioscience), and mouse TrueBlot ULTRA (18‐8817, 1:2000, eBioscience). All antibodies were purchased, and stored according to the manufacturers’ instruction, and used for immunoblotting. Membranes were developed with ECL western blotting substrate (32106, Thermo Fisher Scientific) for 1 min and visualized using LAS4000 (GE Healthcare).

### 4.19 Proximity Ligation Assay (PLA)

Anti‐VEGFR2 antibody (SAB4501645, Sigma–Aldrich) was converted to PLA probes by conjugation to 5´thiolated oligonucleotides as described earlier to detect VEGFR2 homodimers [56]. Briefly, 50 µg monoclonal antibodies were reacted with 30‐fold excess of sulfo‐SMCC (sulfosuccinimidyl 4‐[N‐maleimidomethyl]cyclohexane‐1‐carboxy‐late, Pierce Biotechnology). The reaction product was passed through a 0.2‐µm filtering unit and the unbound sulfo‐SMCC was separated on a Bio‐Rad BioGel P‐30 fine‐size exclusion purification resin (Invitrogen, CA) that had been pre‐equilibrated in PBS. At a ratio of one antibody to four oligonucleotides, the freshly reduced thiolated oligonucleotides were coupled overnight. The PLA probes were then purified by gel filtration, removing unreacted antibodies and oligonucleotides and were then used for tissue staining. To detect Doppel, slides were incubated with Mouse anti‐Doppel and Rabbit anti‐Doppel antibodies for detecting Doppel and thereafter incubated with PLA secondary antibodies pre‐conjugated to unique oligonucleotides. Ligation of the oligonucleotides was followed by an amplification step. The products were detected by using a complementary fluorescently labeled probe. Cells were further stained with FITC labeled phalloidin. Slides were mounted using mounting media. Z‐stack micrographs were taken and evaluated using the confocal microscope (Carl Zeiss LSM710, Germany). The number of homodimers, visualized as red dots, were counted in 10–15 fields/slide. The experiments were repeated at least three times. Cell images were exported using the ZEN 2012 software (Carl Zeiss) in TIF format for further analysis and determination of homodimers/cell in Image J software (NIH).

### 4.20 Doppel cDNA Transfections in Human Endothelial Cells

Generation of Doppel‐expressing stable HUVEC cell line was reported in the previous study [11]. For transient transfection in HDMEC, cells were plated in 60 mm dishes (0.7 x 10^5^ cells per dish) and transfected at day 1 of culture. Briefly, Doppel cDNA (RG223341, Origene) was re‐suspended in 100 µL of RNase‐free water. For each transfection, 0.5 µg of Doppel cDNA and mock cDNA was diluted with 145 µL OptiMEM (51985, Gibco) in different tubes. In another tube, 5 µL of lipofectamine 2000 (Invitrogen) was diluted with 145 µL of OptiMEM. The cDNA solution was directly added to the diluted transfection reagent and the mixture was incubated for 20 min at RT. The mixture was added in each well, which already contains 0.7 mL OptiMEM media. In a separate control well, 5 µL of lipofectamine 2000 was also added without cDNA. 30 min after transfection, 1.0 mL of normal growth media was added. The transfection was allowed to proceed for 24 h before the cells were subjected to subsequent experiments. The expression of Doppel was evaluated from the whole cell lysates of Doppel‐transfected with non‐transfected endothelial cells.

### 4.21 Knockdown of Doppel in Endothelial Cells

For different experiments, Hu^Dpl^ were seeded in 60‐mm plates and transfected using either human Doppel esiRNA (EHU154061; Sigma–Aldrich) or PRND Trilencer siRNA (SR308413, Origene Inc.) following the manufacturer's instructions. TECs isolated from SCC7 and CT26 tumors were transfected using mouse Doppel shRNA (sc‐42205‐SH, Santa Cruz Biotechnology) following the manufacturer's instructions.

### 4.22 Statistical Analysis

We have analyzed normally distributed data using paired, two‐tailed Student's *t*‐test for two‐group comparisons or one‐way ANOVA for multiple comparison tests (GraphPad Prism 6.0, CA). The data are presented as mean ± SD; *p*‐value < 0.05 was considered statistically significant.

## Author Contributions

B.K., H.K.L., J.U.C., I.S.K., S.Y.K., S.W.K., Y.B., and T.A.A. contributed to conceptualization. B.K., H.K.L., Z.A., R.W., N.K.L., W.S.S., and K.T. carried out the investigation. Methodology was developed by B.K., H.K.L., Z.A., R.W., F.A., and T.A.A. Resources were provided by Y.G.K., S.Y.C., and S.R.L. Visualization was performed by B.K., H.K.L., and Z.A. Supervision was undertaken by I.S.K., Y.B., and T.A.A. B.K., H.K.L., and T.A.A. wrote the original draft of the manuscript. Writing – review and editing was performed by Y.B. and T.A.A. Project administration was managed by S.Y.K., Y.B., and T.A.A. Funding acquisition was carried out by S.Y.K., Y.B., and T.A.A.

## Funding

This study was supported by grants from NIH R21CA264627, NIH R01CA262788, NIH SC1GM144171, DoD HT94252410217, DoD STTR Phase II #HT942523C0044, Lizanell and Colbert Coldwell Foundation #NAID20220187 that are awarded to Dr. Taslim Al‐Hilal. Korea Drug Development Fund by Ministry of Science and ICT grant No. HN21C0264 (YB). Ministry of Trade, Industry, and Energy, and Ministry of Health and Welfare; the National Research Foundation of Korea (NRF) funded by the Korea government (MSIT) grant No. 2020R1A2C2015026 (YB) BK21 FOUR program of the National Research Foundation of Korea (NRF) funded by the Ministry of Education (BK, HKL). The study was also supported by 2025 The Hur Jiyoung Foundation (to BK). 2022 Health Fellowship Foundation (to HKL).

## Conflicts of Interest

The authors declare no conflicts of interest.

## Supporting information




**Supporting File**: advs73917‐sup‐0001‐SuppMat.docx.

## Data Availability

The data that support the findings of this study are available from the corresponding author upon reasonable request.
