# Supplementary material for: Selective Targeting of Tip Endothelial Cells as a Therapeutic Strategy for Tumor Angiogenesis
Source: Adv Sci (Weinh). 2026 Feb 15;13(18):e12975. doi: 10.1002/advs.202512975 (PMC13042361; doi:10.1002/advs.202512975)
Supplement: Supplementary file 1 — Supporting File: advs73917‐sup‐0001‐SuppMat.docx. [file ADVS-13-e12975-s001.docx]

Supporting Information

Selective targeting of tip endothelial cells as a therapeutic strategy for tumor angiogenesis

Byoungmo Kim^1†^, Ha Kyeong Lee^1†^, Zulfikar Azam^2^, Jeong Uk Choi^3^, Riajul Wahab^2^, Na Kyeong Lee^4^, Yoon Gun Ko^5^, So-Young Choi^6^, Se-Ra Lee^6^, Wan Seob Shim^1^, Taeeung Kim^1^, In-San Kim^7^, Farzana Alam^8^, Sang Yoon Kim^5^, Seong Who Kim^9^, Youngro Byun^1,5^*, Taslim A Al-Hilal^3^*

E-mail address: yrbyun@snu.ac.kr (Y. Byun), taslim.alhilal@pharm.utah.edu (T.A. Al-Hilal)


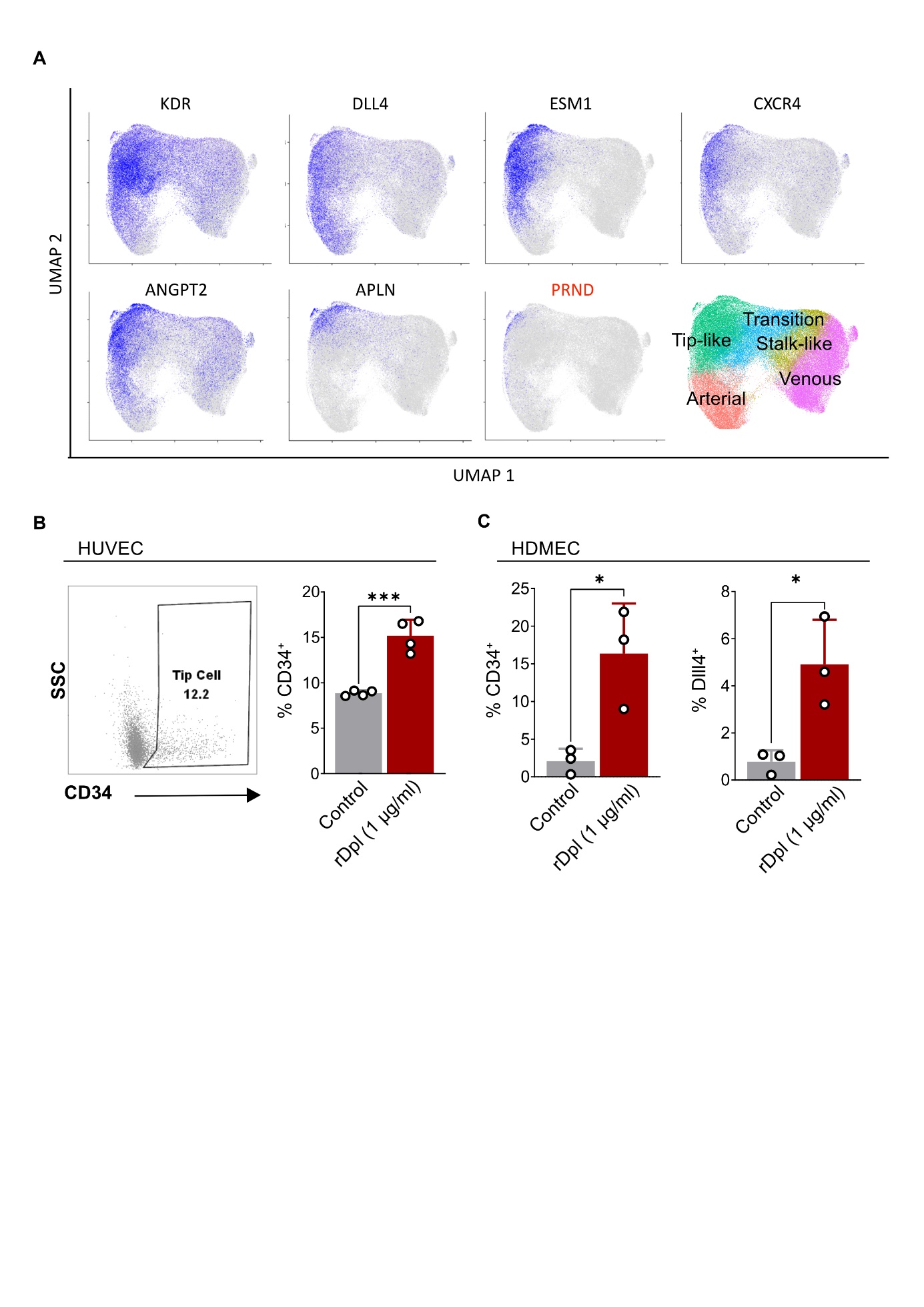


**Figure S1. PRND is selectively expressed in tip endothelial cells.**

**(A)** Expression of known tip cell markers overlaid on the UMAP projection. PRND expression is highly selective towards tip cells. **(B)** Flow cytometry gating strategy to identify CD34⁺ ^Tip^ECs in HUVEC. Right panel: Quantification of CD34⁺ cells after 24 h treatment with recombinant Doppel (rDpl, 1 µg mL^-1^). Dots represent independent experiments, *n* = 3 per condition. **(C)** Quantification of CD34⁺ and Dll4^+^ cells in HDMEC after 24 h treatment with recombinant Doppel (rDpl, 1 µg mL^-1^). Dots represent independent experiments, *n* = 3 per condition. All data are presented as mean ± standard deviation (SD). Two-tailed Student’s t test; *P****<0.001, *P**<0.05.

**
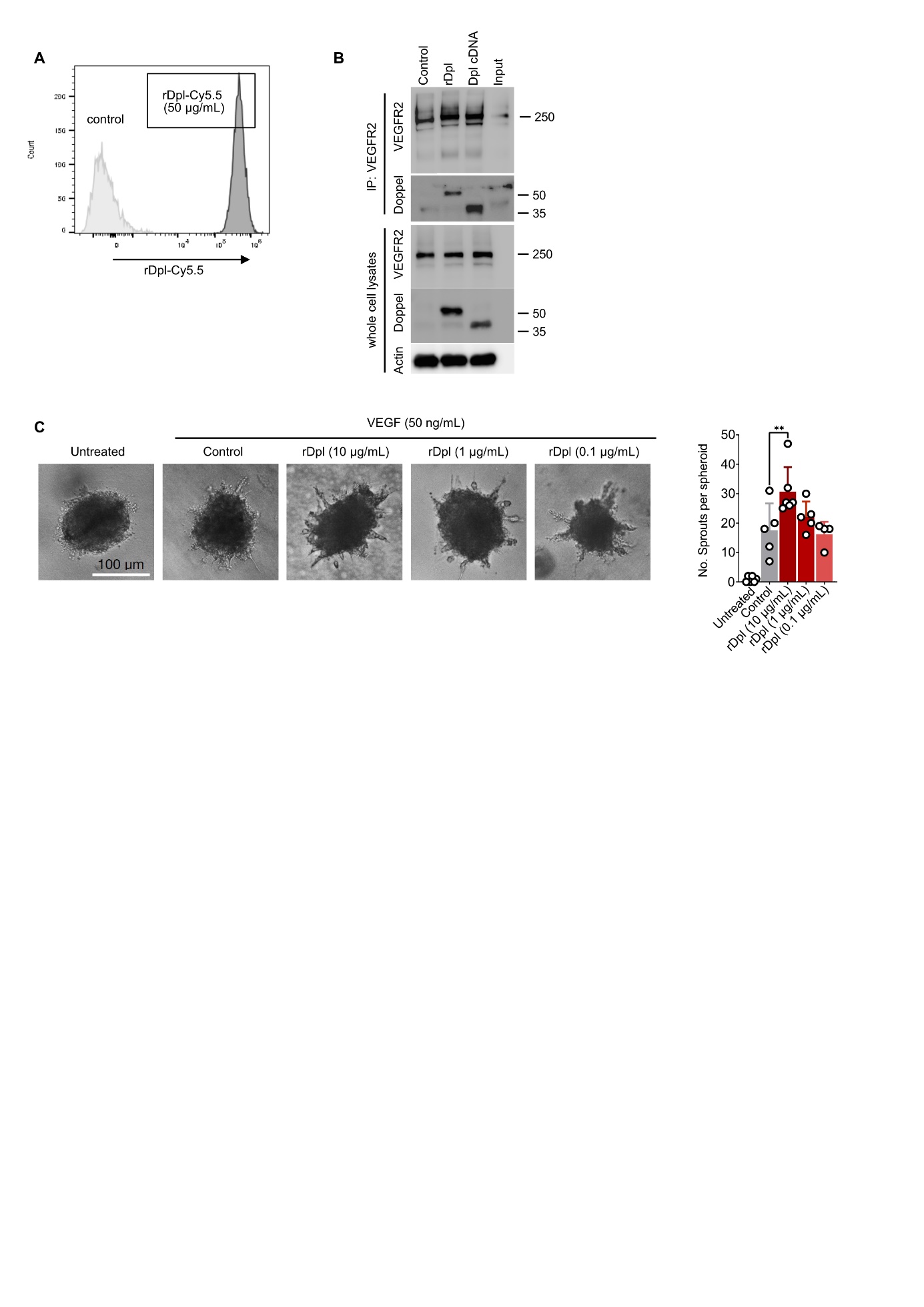
**

**Figure S2. Recombinant Doppel binds to endothelial cells and enhances VEGFR2 signaling and angiogenic sprouting.**

**(A)** Flow cytometric analysis of Cy5.5-labeled recombinant Doppel (rDpl) binding to human umbilical vein endothelial cells (HUVECs). HUVECs were incubated with Cy5.5-rDpl, and surface binding was quantified based on Cy5.5 fluorescence intensity. Histogram depicts a clear shift in fluorescence relative to unstained control, indicating surface-specific binding of rDpl to endothelial cells. **(B)** Immunoprecipitation followed by western bot (IP-WB) for VEGFR2 and Doppel in the total cell lysates of HUVEC, HUVEC treated with 10 µg mL^-1^ recombinant Doppel, and Doppel cDNA transfected HUVEC cells. Whole cell lysates of each respective group were also blotted as control. **(C)** Representative images of VEGF-induced endothelial sprouting from HUVEC spheroids treated with increasing concentrations of rDpl. White asterisks indicate HUVEC sprouts. Scale bar, 100 μm. Right panel: Quantification of sprouts per spheroid. *n* = 5 experimental replicates; 50 spheroids per condition. Data are presented as mean ± standard deviation (SD). Statistical analysis was performed using one-way ANOVA followed by Tukey’s multiple-comparison test; *P***< 0.01, nonsignificant comparisions not shown.


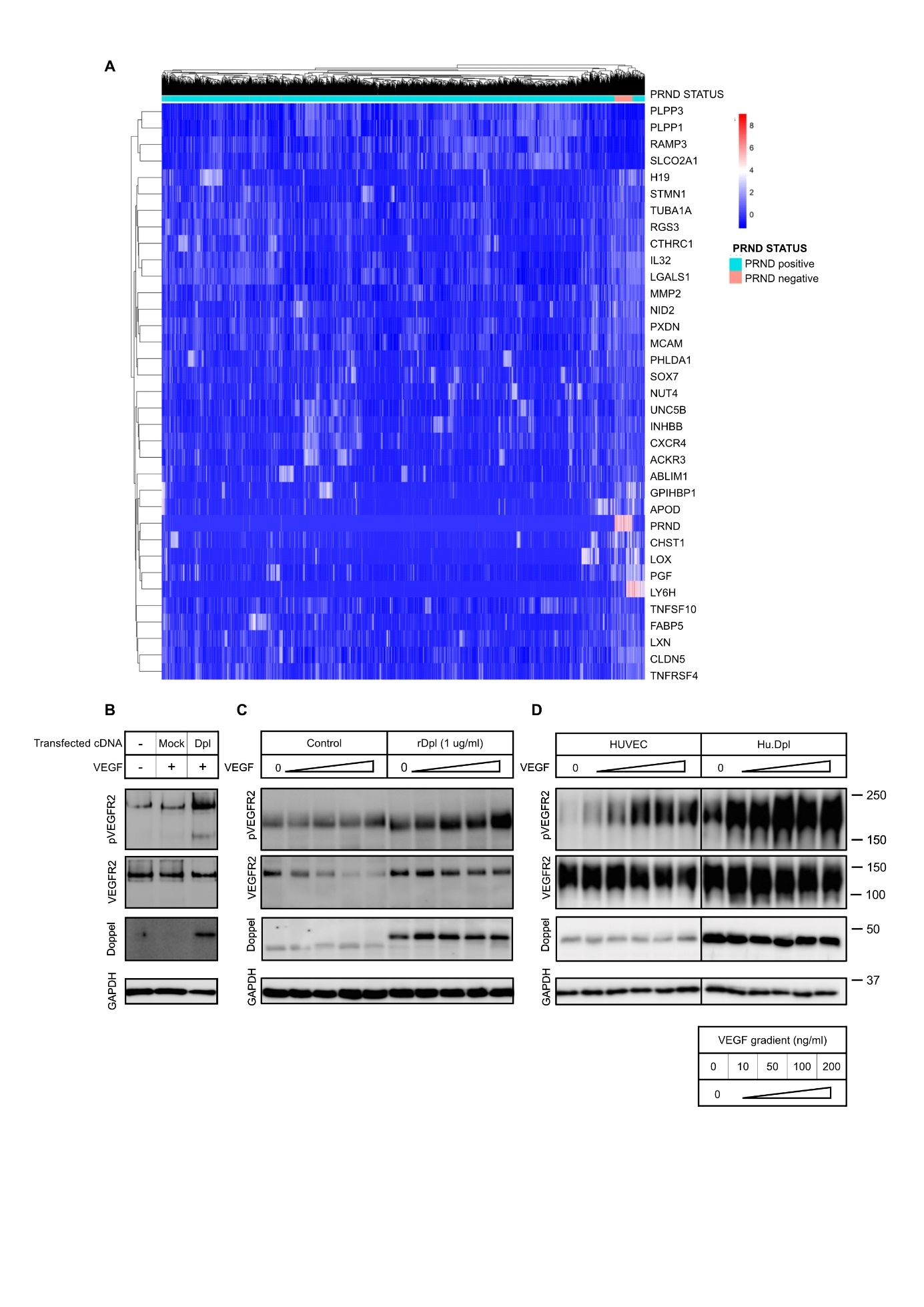


**Figure S3. Doppel increases VEGFR2 signaling**

**(A)** Heatmap showing scaled expression (Z-score) of the top 40 differentially expressed genes between PRND⁺ and PRND⁻ endothelial cells. Rows represent genes, and columns represent individual cells. Gene expression was normalized and scaled per gene. Color scale represents relative gene expression (blue = low; red = high). **(B)** Western blot analysis for phosphorylated VEGFR2 (pVEGFR2), VEGFR2, Doppel and GAPDH in the total cell lysates of HUVEC, as well as HUVEC cells transfected with either mock (empty vector control) or Doppel cDNA treated with 100 ng mL^-1^ of VEGF-A. Doppel protein levels confirm knockdown efficiency. **(C)** Western blot analysis for phosphorylated VEGFR2 (pVEGFR2), VEGFR2, Doppel and GAPDH in HUVECs treated with increasing doses of VEGF (0, 10, 50, 100, 200 ng mL^-1^ ) in the presence or absence of Fc-tagged rDpl (arrow, 1 µg mL^-1^). GAPDH was used as a loading control. **(D)** Western blot for pVEGFR2, VEGFR2, Doppel, and GAPDH in HUVEC and Hu.Dpl cells treated with serial gradients of VEGF (0, 10, 50, 100, 200 ng mL^-1^). Doppel expression was validated with anti-Doppel antibody.


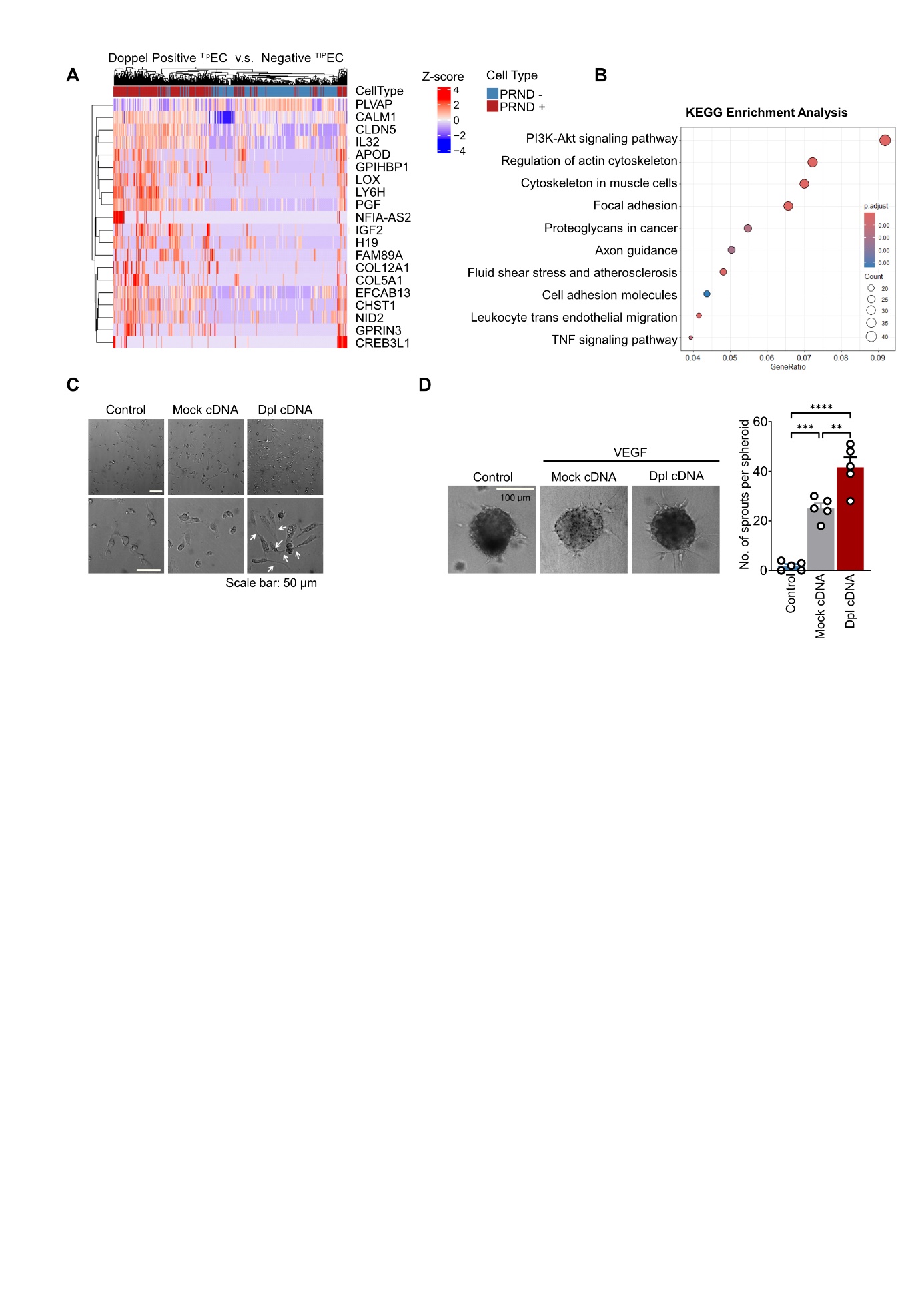


**Figure S4. Doppel promotes tip cell morphology and cytoskeletal remodeling.**

**(A)** Heatmap of the top 20 differentially expressed genes (DEGs) PRND⁺ and PRND⁻ ^Tip^ECs. A total of 1000 randomly selected cells per group were analyzed. Rows represent genes; columns represent individual cells. Expression values were log-normalized and Z-scored. Color scale: red = high expression; blue = low expression. **(B)** KEGG enrichment analysis of DEGs in PRND^+^ versus PRND^-^ ^Tip^ECs. Dot size indicates gene count; color represents adjusted *P* value. **(C)** Representative high and low magnification brightfield image of mock- or Doppel-transfected HDMECs. White arrows denote filopodia. Scale bar 50 μm. **(D)** Representative brightfield image of mock- or Doppel-transfected HDMEC spheroids. Scale bar, 100 μm. Right panel: Quantification of sprouts per spheroid. *n* = 5 experimental replicates; 50 spheroids per condition. Data are presented as mean ± SD. Statistical analysis was performed using one-way ANOVA followed by Tukey’s multiple-comparison test; *P*****<0.0001, *P****<0.001, *P***< 0.01.


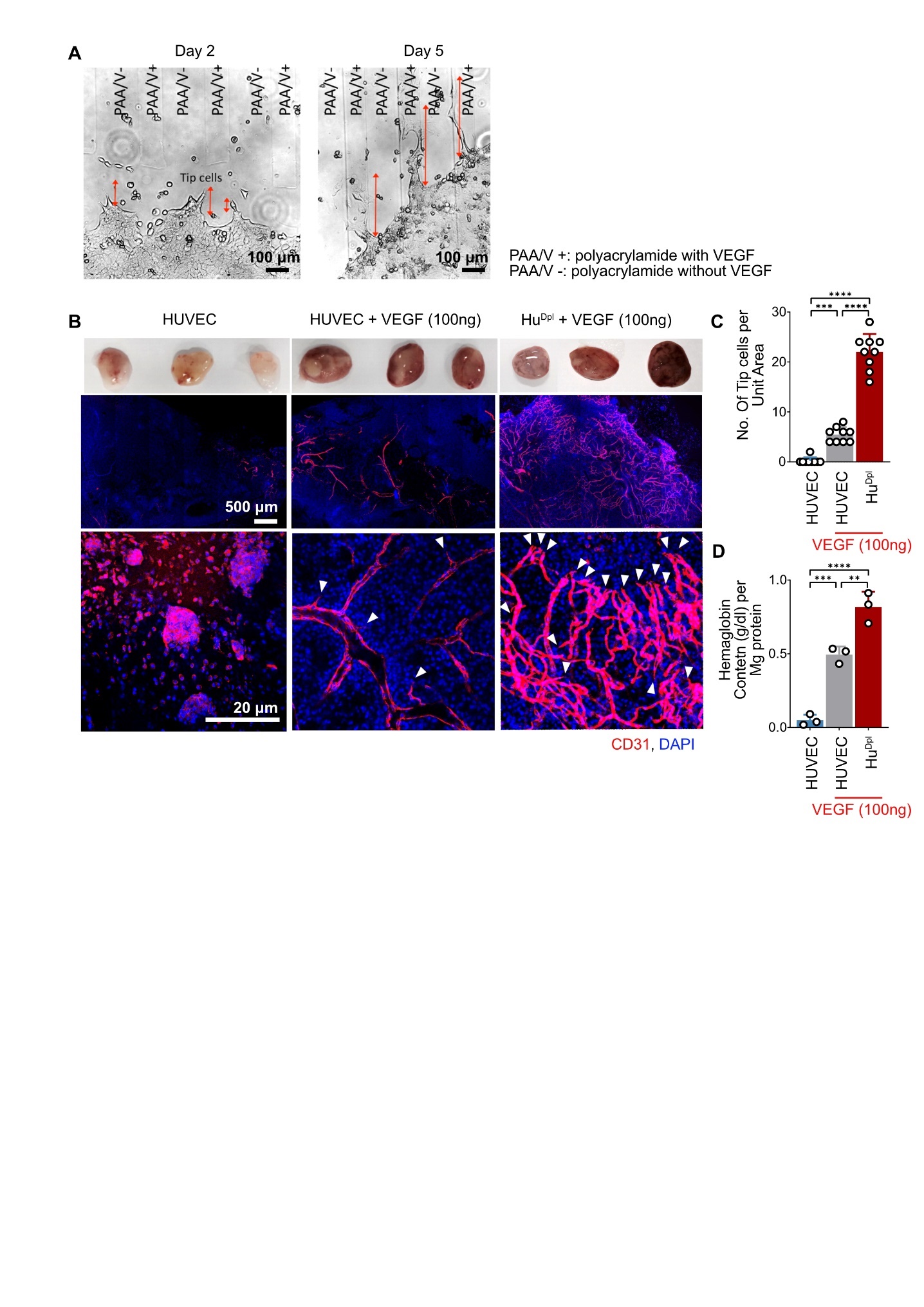


**Figure S5. Doppel enhances tip cell formation and perfused vessel growth in vivo.**

**(A)** Representative brightfield images of Hu^Dpl^ spheroid sprouting along the V+ stripe, scale bar, 100μm **(B)** Representative high- and low-magnification confocal images of whole-mount Matrigel plugs containing HUVEC or Hu^Dpl^ spheroids, stained for CD31 and DAPI. Spheroids were embedded in Matrigel and incubated in the presence or absence of VEGF (100 ng). White arrows indicate tip cells. CD31, red; DAPI, blue. Scale bars: 500 μm (top), 20 μm (bottom). **(C)** Number of observed tip cells per frame of Matrigel plugs. Dot represents areas quantified from three plugs. **(D)** Blood content denoted as hemoglobin content within Matrigel plug according to impregnated spheroids. Dot represents individual plug. All data are presented as mean ± standard deviation (SD). Statistical analysis was performed using one-way ANOVA followed by Tukey’s multiple-comparison test; *P*****<0.0001, *P****<0.001, *P***< 0.01.


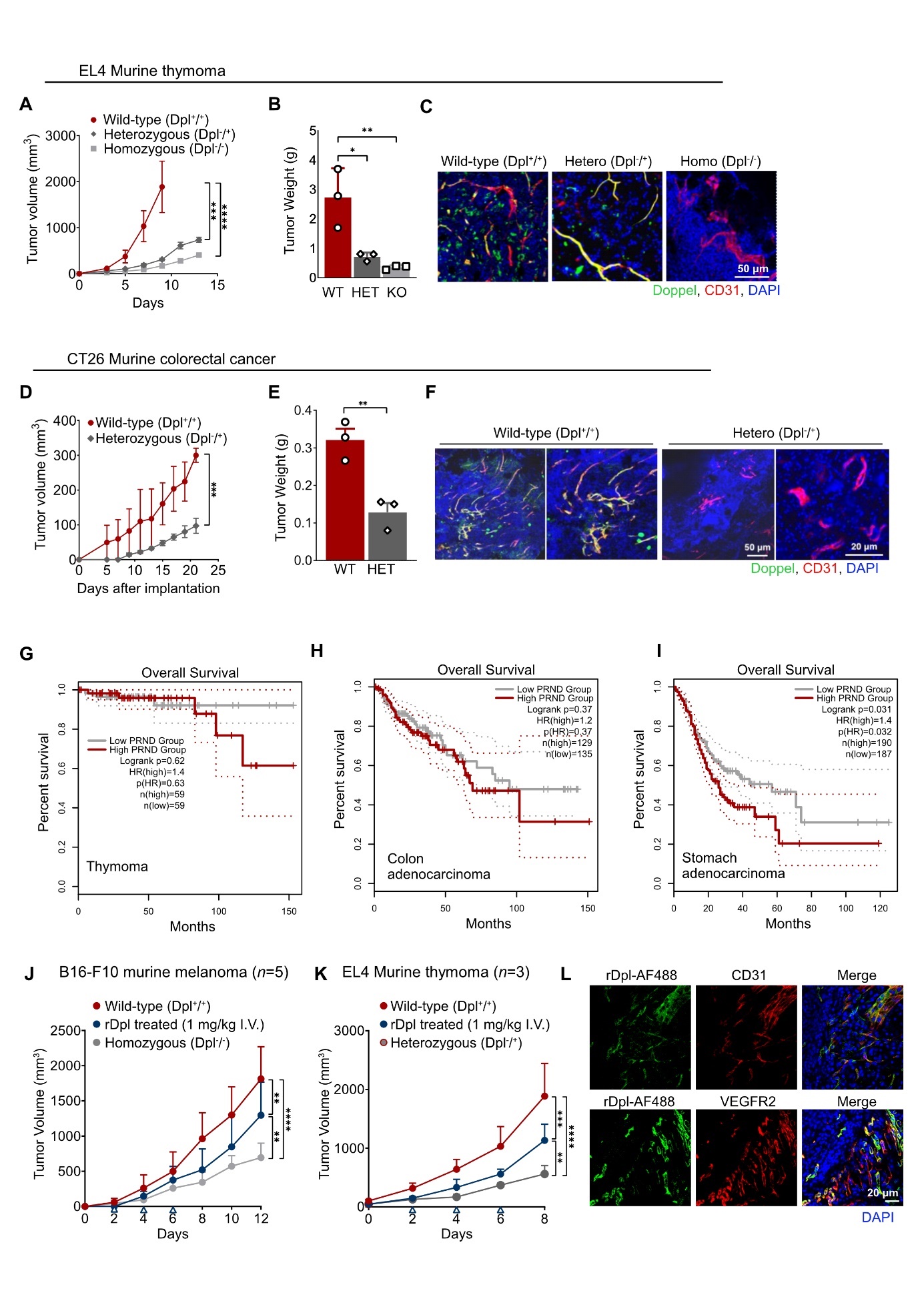


**Figure S6.** **Tumor growth is highly dependent on Doppel expression**

**(A)** Tumor growth curves of EL4 thymoma tumors in wild-type (Dpl⁺/⁺), heterozygous (Dpl⁺/⁻), and homozygous Doppel knockout (Dpl⁻/⁻) C57BL/6 mice over 13 days post-inoculation, *n* = 3. Statistical comparisions were for tumor volumes on day 9. **(B)** Tumor weights at endpoint. One-way ANOVA followed by Tukey’s multiple-comparison test. **(C)** Representative immunofluorescent images showing vessel density and Doppel expression in tumor tissues from mice of each genotype. Doppel, green; CD31, red; DAPI, blue. Scale bar, 20 μm. **(D)** Tumor growth curves of CT26 colorectal cancer in wild-type (Dpl⁺/⁺) and heterozygous (Dpl⁺/⁻) Doppel knockout C57BL/6 mice over 21 days post-inoculation, *n* = 3. Two-way ANOVA followed by Tukey’s multiple-comparison test; P***< 0.001.  **(E)** Tumor weights at endpoint, Two-tailed Student’s t test; P**< 0.01. **(F)** Representative immunofluorescent images showing vessel density and Doppel expression in tumor tissues from mice of each genotype. Doppel, green; CD31, red; DAPI, blue. Scale bar, 50 μm (left), 20 μm. **(G to I)** Kaplan–Meier survival curves for thymoma **(G)**, colorectal adenosarcoma **(H)**, and stomach adenosarcoma **(I)** patients stratified by *PRND* expression levels. Analysis was performed using GEPIA2 based on TCGA datasets. **(J)** Tumor growth curves in WT and Doppel-knockout (KO) mice bearing B16F10 murine melanoma tumors, with or without recombinant Doppel treatment at 1 mg kg^-1^, *n* = 5. Two-way ANOVA followed by Tukey’s multiple-comparison test. **(K)** Tumor growth curves in WT and Doppel-heterozygous mice bearing EL4 murine thymoma tumors, with or without recombinant Doppel treatment at 1 mg kg^-1^, *n* = 3. Two-way ANOVA followed by Tukey’s multiple-comparison test. **(L)** Images of fluorescently labeled recombinant Doppel binding to VEGFR2 on CD31+ blood vessels. rDpl-AF488, green; CD31 (top) or VEGFR2 (bottom), red; DAPI, blue. Scale bars: 20 μm. All data are presented as mean ± standard deviation (SD). Arrows indicate treatment schedule of rDpl. Two-way ANOVA followed by Tukey’s multiple-comparison test; P****<0.0001, P***<0.001, P**< 0.01, nonsignificant comparisons not shown.


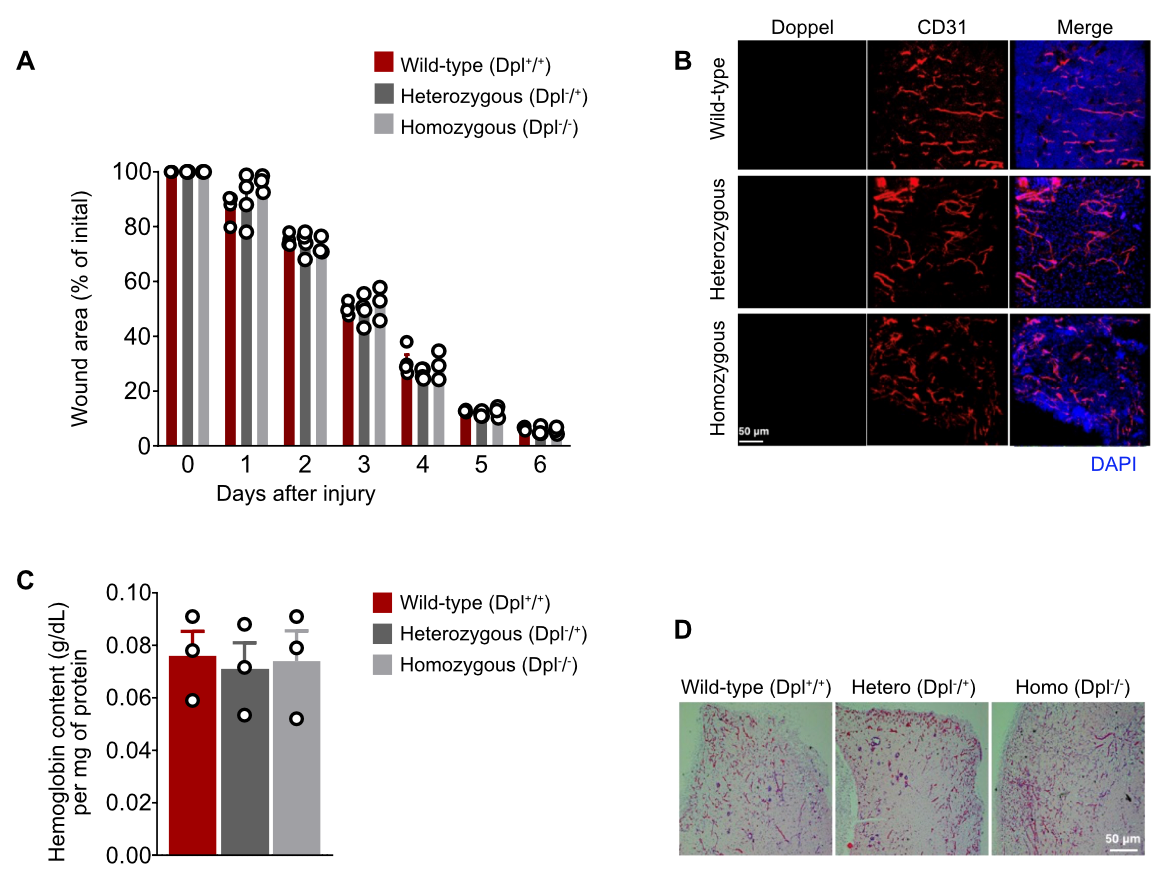


**Figure S7.** **Doppel deficiency does not impair wound healing and angiogenic sprouting in Matrigel plugs in vivo.**

**(A)** Quantification of wound healing kinetics in wild-type, heterozygous, and homozygous Doppel-deficient mice following full-thickness excisional injury. Wound closure was monitored daily, showing no difference in re-epithelialization in Doppel-deficient mice compared with wild-type controls, *n* = 3. **(B)** Representative immunofluorescent images showing vessel density and Doppel expression in healed wound tissue from mice of each genotype. Doppel, green; CD31, red; DAPI, blue. Scale bar, 50 μm. **(C)** Quantification of hemoglobin content in Matrigel plugs, presented as g/dL per mg of total protein, to estimate functional vascularization. No significant differences were observed among the three groups *n* = 3 **(D)**. Representative images of vascular structures in Matrigel plugs implanted subcutaneously into wild-type (Dpl⁺/⁺), heterozygous (Dpl⁺/⁻), and homozygous Doppel knockout (Dpl⁻/⁻) C57BL/6 mice. Plugs were harvested and stained to visualize vessel density. Scale bar, 50 μm. Data are presented as mean ± SD. No statistically significant differences were observed among the groups.


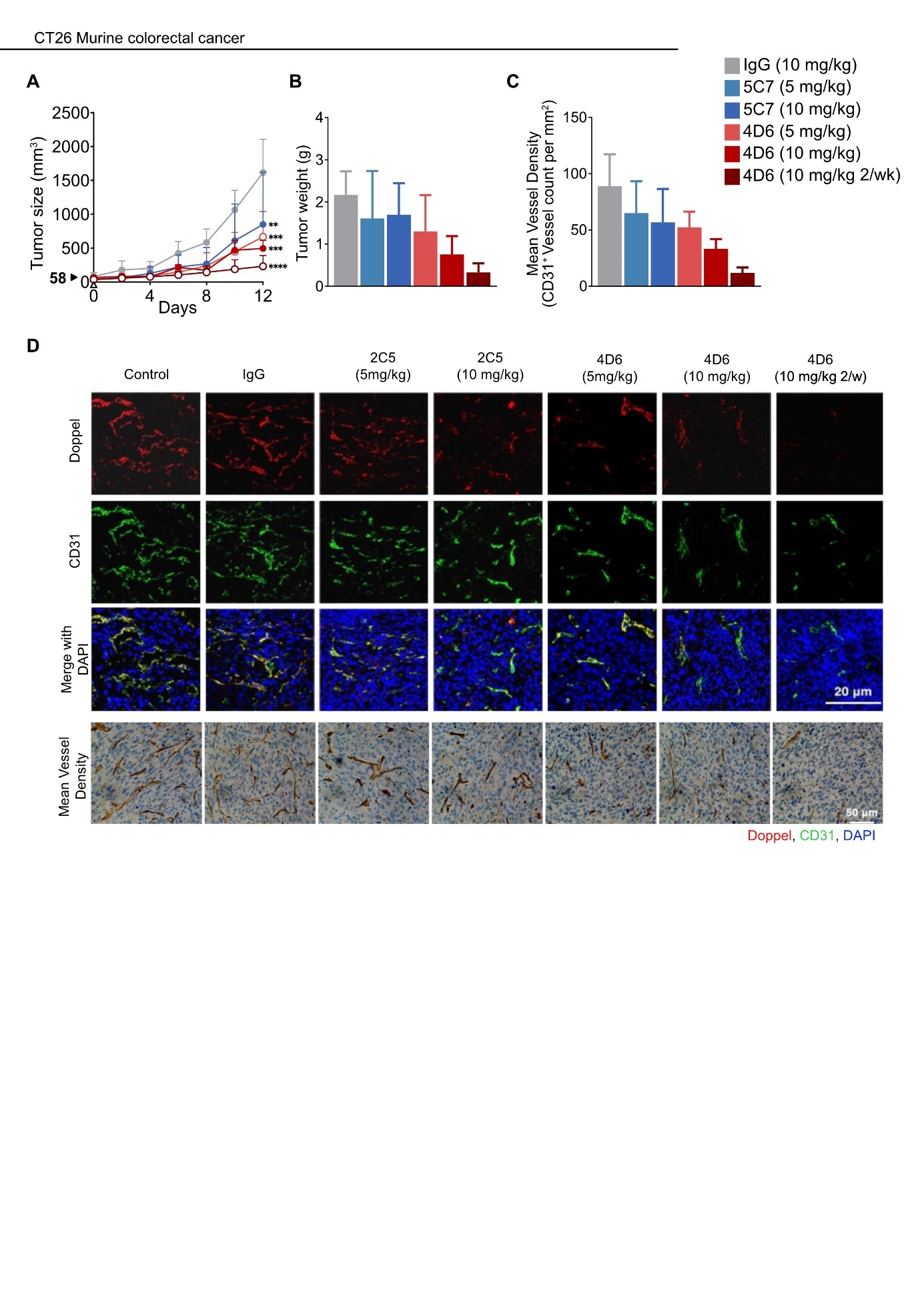


**Figure S8. Doppel-blocking antibodies reduces vascular density and suppresses CT26 tumor growth**

**(A)** Tumor growth curves of CT26 colorectal tumors treated with human IgG isotype control and anti-Doppel antibodies: 5C7 (blue) and 4D6 (red) at 10 mg kg^-1^, *n* = 4-5. Two-way ANOVA followed by Tukey’s multiple-comparison test; *P***< 0.01, *P****< 0.001 *P*****< 0.0001. **(B)** Tumor weights at endpoint, pooled from two independent experiments. **(C)** Quantification of mean vessel density based on CD31 staining. Data represents vessel counts per unit area. **(D)** Representative immunofluorescence of CT26 tumor sections for staining with different markers. Doppel, red; CD31, green; DAPI, blue. Scale bar, 20 μm. CD31 immunohistochemical staining is also shown. Scale bar, 50 μm.


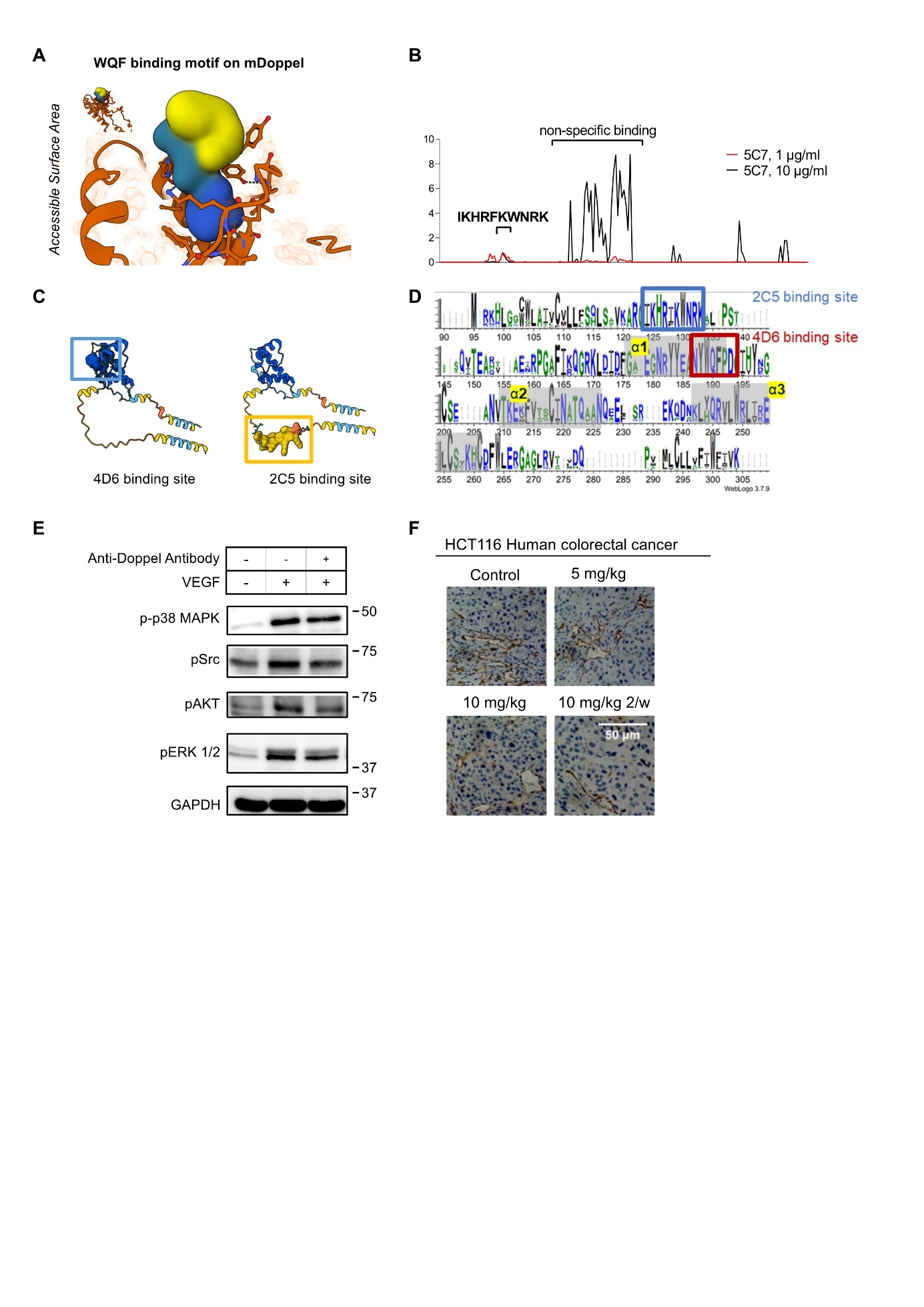


**Figure S9. Binding motif characterization and structural conservation of 4D6 and 5C7 antibodies targeting Doppel.**

**(A)** Surface accessibility rendering of the WQF motif from murine Doppel (AlphaFold model: AF-Q9QUG3-F1-v4). Solvent-exposed surface calculated in Mol*. Color bar denotes solvent-accessible surface area. **(B)** PEPperMAP^®^ epitope mapping of murine Doppel using monoclonal antibody 5C7 at 1 µg mL^-1^and 10 μg mL^-1^. At 1 μg mL^-1^, 5C7 showed two weak but reproducible responses to peptides containing the consensus motifs HLSTVKARGIK and IKHRFKWNRK. At 10 μg mL^-1^, nonspecific background signal increased, with additional binding to peptides rich in aromatic residues (e.g., EGNRY, FPDGIY), likely representing off-target or low-affinity interactions. **(C)** Structural modeling of the 4D6 and 5C7 binding epitopes on the AlphaFold-predicted human Doppel structure (AF-Q9UKY0-F1-v4). Protein cartoon is color-coded by pLDDT confidence scores (blue: high; yellow/red: low). Antibody binding sites are highlighted in blue (4D6) and yellow (5C7) boxes. **(D)** Sequence conservation analysis of Doppel epitope regions across 282 eukaryotic species. WebLogo3 output of multiple sequence alignment using Clustal Omega, based on sequences retrieved from OrthoDB (ortholog group 9523143at2759). Letter height denotes information content (bits); grey bars represent standard deviation. The 4D6 (YWQFPD) and 5C7 (IKHRFKWNRK) binding motifs are boxed in red and blue, respectively. Predicted α-helical domains (α1, α2, α3) are indicated in grey shading. **(E)** Western blot analysis of VEGF-stimulated HUVEC cells treated with or without anti-Doppel antibody. Inhibition of Doppel suppressed VEGF-induced phosphorylation of p38 MAPK, Src, AKT, and ERK1/2, indicating that Doppel blockade attenuates key angiogenic signaling pathways downstream of VEGFR2. GAPDH served as the loading control. **(F)** CD31 immunohistochemical staining images of HCT116 tumor sections of mice treated with different concentrations of 4D6. Scale bar, 50 μm. All data are presented as mean ± standard deviation (SD).


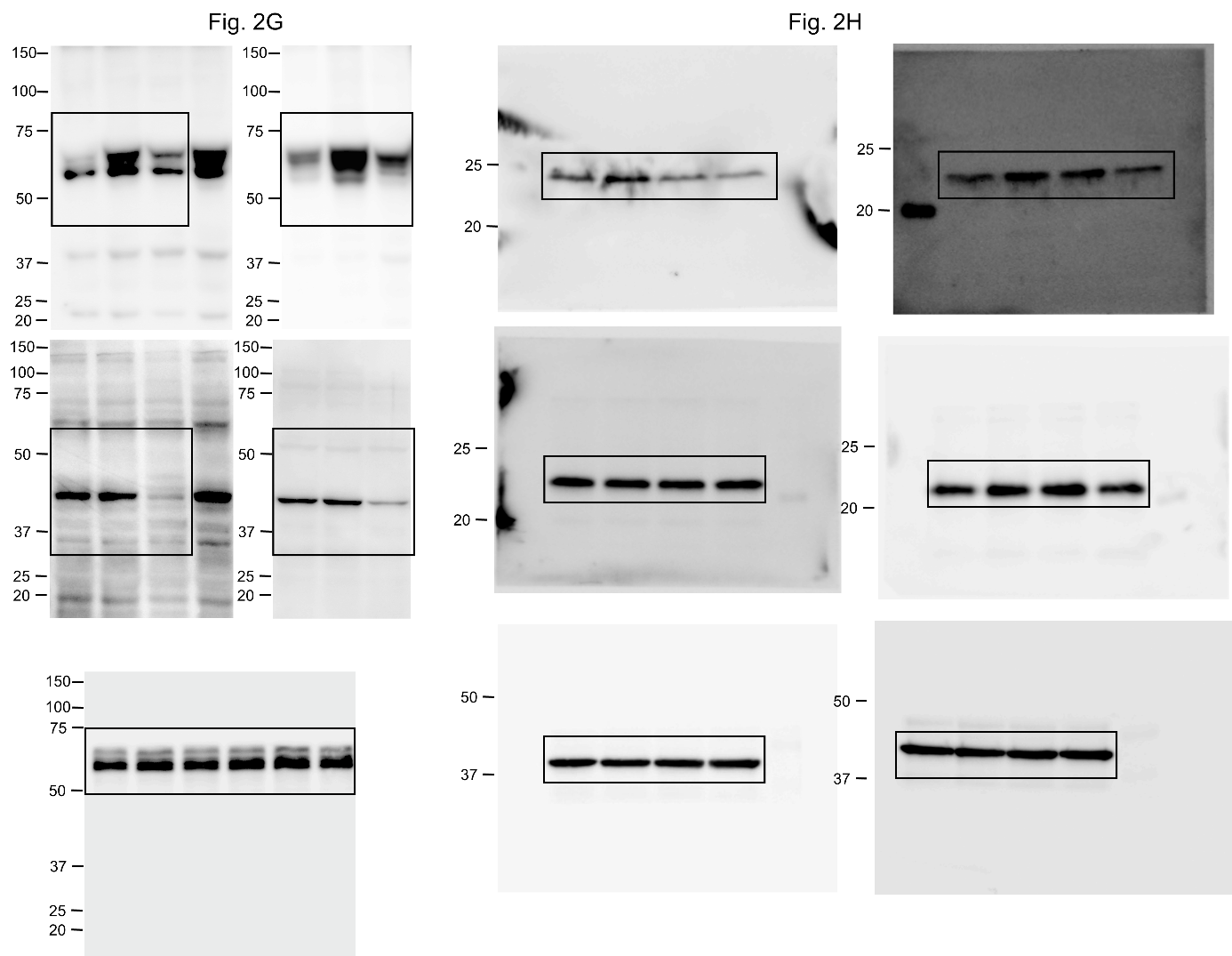


**Figure S10. Unprocessed blots for Figure 2G, 2H.**

**
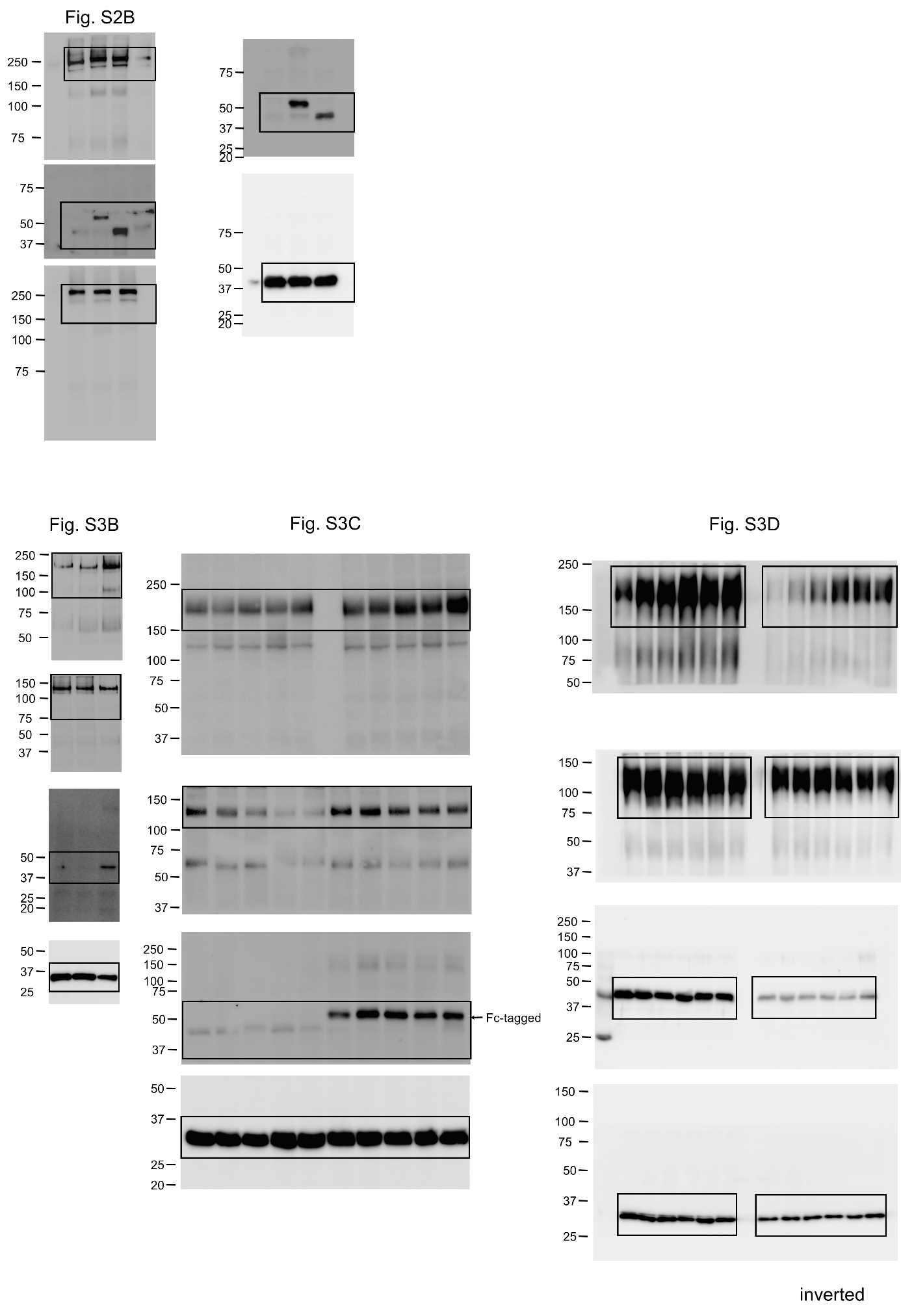
**

**Figure S10. Unprocessed blots for Figure S2B.**

**
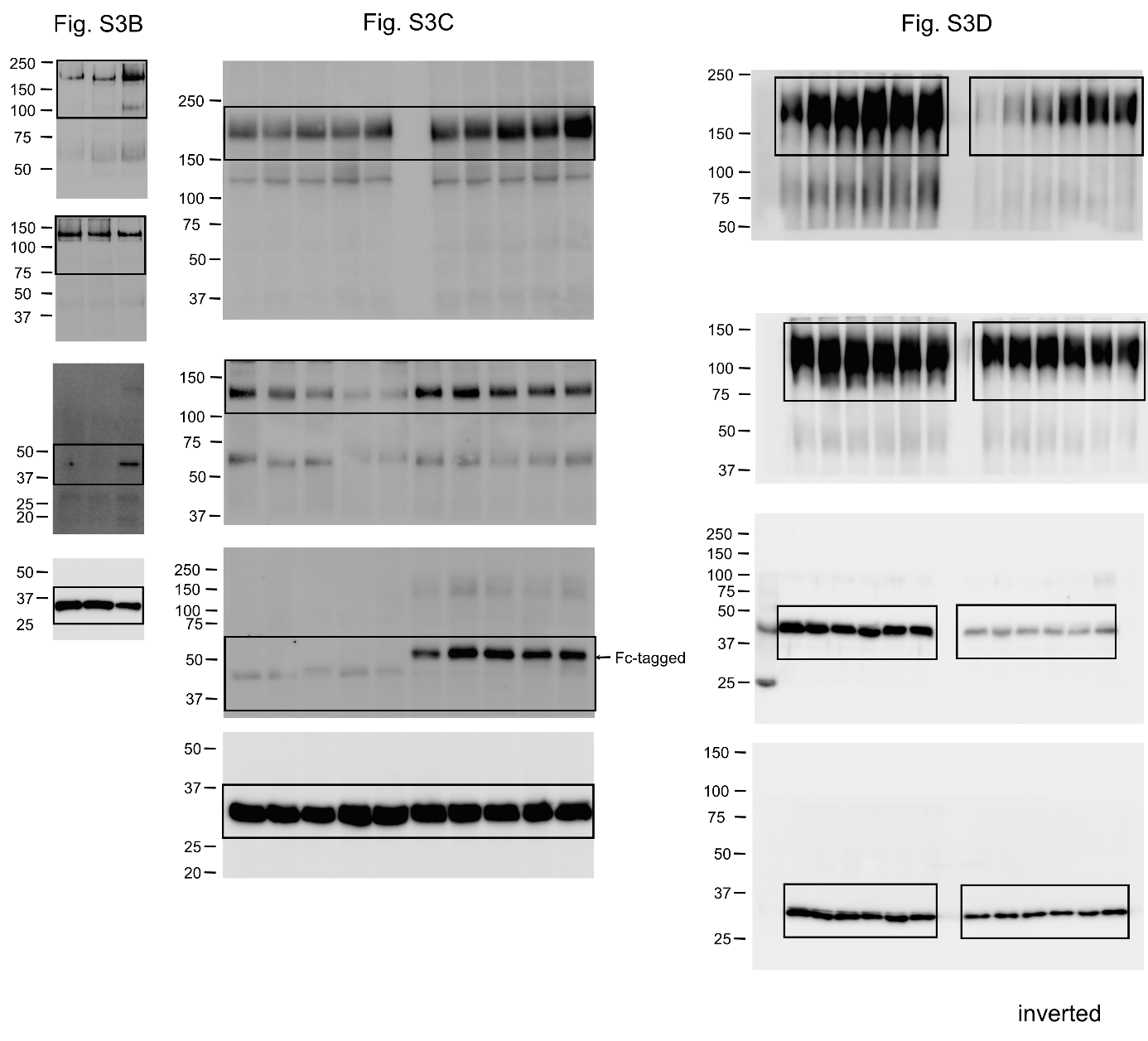
**

**Figure S10. Unprocessed blots for Figures 3B, 3C, 3D. (3D)** In the main figure, the left and right Western blot panels were rearranged to improve clarity and facilitate comparison

**
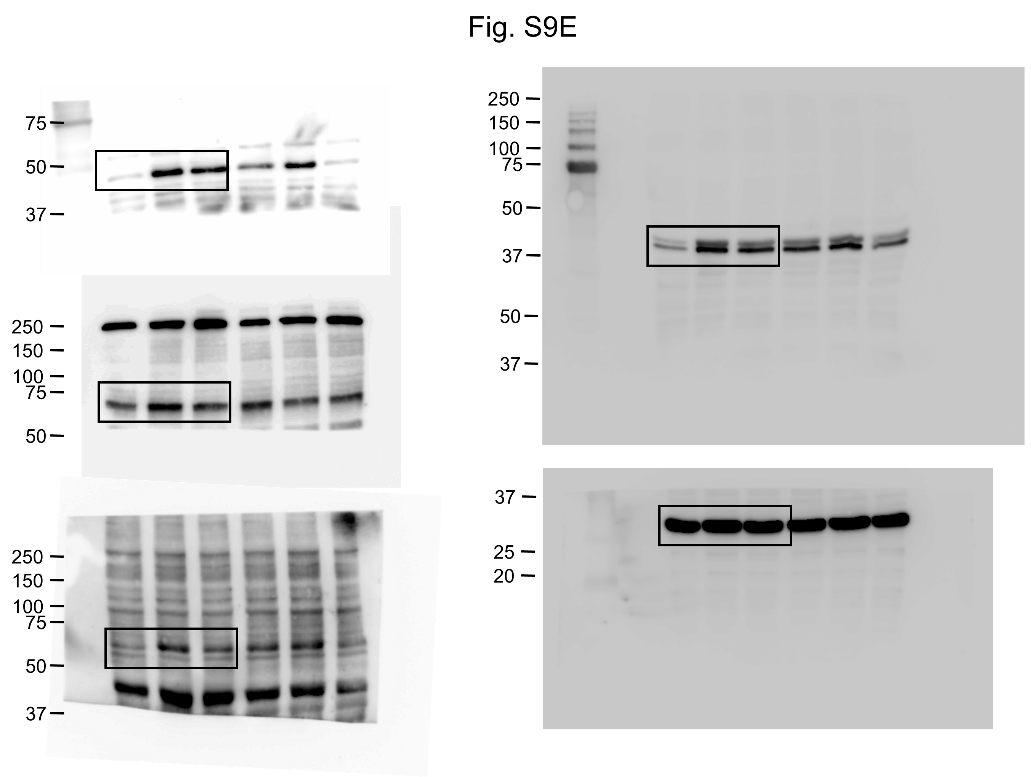
**

**Figure S10. Unprocessed blots for Figure S9E.**
